# Identification of natural TLR4 modulators through network pharmacology and molecular modeling in SARS-CoV-2 *Acinetobacter baumannii* co-infection

**DOI:** 10.3389/fchem.2026.1916568

**Published:** 2026-09-16

**Authors:** Mebarka Ouassaf, Shafi Ullah Khan, Kannan R. R. Rengasamy, Nada S. Alhaggass, Bader Y. Alhatlani

**Affiliations:** 1 Group of Computational and Medicinal Chemistry, LMCE Laboratory, University of Biskra, Biskra, Algeria; 2 Independent Researcher, Hérouville-Saint-Clair, France; 3 Laboratory of Natural Products and Medicinal Chemistry (LNPMC), Saveetha Dental College and Hospital, Saveetha Institute of Medical and Technical Sciences (SIMATS), Chennai, India; 4 Centre of Excellence for Pharmaceutical Sciences, North-West University, Potchefstroom, South Africa; 5 Department of Biology, College of Science, Qassim University, Buraydah, Saudi Arabia; 6 Unit of Scientific Research, Applied College, Qassim University, Buraydah, Saudi Arabia

**Keywords:** *Acinetobacter baumannii*, admet, co-infection, host-directed therapy, molecular docking, molecular dynamics, natural compounds, network pharmacology

## Abstract

Viral–bacterial co-infection with SARS-CoV-2 and *Acinetobacter baumannii* exacerbates hyperinflammation via Toll-like receptor 4 (TLR4)-mediated immune pathways. Using network pharmacology, an integrated protein–protein interaction network identified 30 key inter-species hubs comprising viral, bacterial, and human host proteins, with TLR4 achieving a high MCC score (722), highlighting its high topological centrality as a host immune target. Virtual screen of 2,820 natural compounds against the TLR4/MD-2 complex identified three top candidates, CID5898023, CID5403474, and CID74977829, with docking scores of −10.46, −10.02, and −9.58 kcal/mol, respectively, compared with −9.10 kcal/mol for the reference antagonist Eritoran. CID5898023 (curcumin-derived) exhibited a more favorable docking score and stable binding behavior during the molecular dynamics simulation compared with the reference antagonist Eritoran. CID74977829 (baicalin-derived) showed stable binding but lower intestinal absorption, while CID5403474 (chrysin-derived) lacked conformational stability despite favorable docking scores. These computational findings prioritize curcumin- and baicalin-derived scaffolds as potential TLR4/MD-2 modulators for further experimental investigation in the context of co-infection-associated inflammation.

## Introduction

The convergence of SARS-CoV-2 and multidrug-resistant *Acinetobacter baumannii* has emerged as a formidable threat in contemporary infectious disease dynamics, particularly in intensive care settings where co-infection rates reach up to 28% and *A. baumannii* alone accounts for over 40% of secondary pneumonias in high-burden regions (Rangel et al., 2021; Lucien et al., 2021). This dual infection scenario is not merely additive but synergistically worsens clinical outcomes through complex immune dysregulation: while SARS-CoV-2 suppresses type I interferon (IFN-I) responses—disabling frontline antiviral defenses—*A. baumannii* exploits this vulnerability to colonize host tissues, releasing lipopolysaccharide (LPS) that hyperactivates Toll-like receptor 4 (TLR4) (Znaidia et al., 2022; King and Sprent, 2021). This triggers a catastrophic NF-κB/STAT3-mediated cytokine storm, elevating acute respiratory distress syndrome (ARDS) mortality to 67% versus 38% in monomicrobial infections (Ma et al., 2025).

In the context of viral–bacterial co-infections, the excessive activation of innate immune pathways has been increasingly linked to severe inflammatory damage (Barman and Metzger, 2021). Among these, Toll-like receptor 4 (TLR4) has emerged as a key immunological node involved in sensing both viral components and bacterial endotoxins (Boehme and Compton, 2004). Several phytochemicals—such as curcumin, resveratrol, baicalin, Chrysin and parthenolide—have shown promising TLR4 antagonism in preclinical studies (Dai et al., 2022; Grabarczyk et al., 2024; Inchingolo et al., 2022; Zhang et al., 2024). However, their clinical development is often limited by poor bioavailability, structural instability, toxicity, or low potency (Shehzad et al., 2010; Yu et al., 2022). Despite these advances, the molecular interplay among SARS-CoV-2, *A. baumannii*, and host immune responses remains insufficiently characterized, particularly regarding the shared molecular nodes that may contribute to amplified inflammation during co-infection (Nussinov, 2015). Moreover, identifying highly connected proteins alone does not necessarily establish their suitability as therapeutic targets, highlighting the need for an integrated strategy that combines network topology with biological relevance and structural suitability for ligand-based investigation. Therefore, the present study aimed to construct an integrated host–virus–bacterium interaction network to identify key inter-species hubs and inflammatory pathways associated with co-infection. Network-based target prioritization was subsequently integrated with biological and structural considerations to select a suitable host target for structure-based investigation.

Finally, natural compounds were screened against the prioritized TLR4/MD-2 complex using molecular docking, followed by ADMET profiling and molecular dynamics simulations, to identify potential modulators of co-infection-associated inflammatory signaling. This study was designed as a computational lead-screening investigation, and the predicted binding affinity, pharmacokinetic properties, and molecular stability reported herein should not be interpreted as evidence of confirmed pharmacological efficacy. Experimental validation is therefore required to establish target engagement, anti-inflammatory activity, and biological efficacy.

## Materials and methods

### Network construction and integration

To investigate the molecular interplay underlying co-infection scenarios, a comprehensive protein–protein interaction (PPI) network was constructed to model the synergistic relationships between SARS-CoV-2, *A. baumannii*, and the human host immune response.

Viral–host interaction data were retrieved from the BioGRID COVID-19 Project (version 4.4.246) (Stark et al., 2006), which provides curated SARS-CoV-2–human protein interaction data. To reduce network complexity and focus the analysis on host responses relevant to the co-infection context, the initial network was biologically filtered by retaining SARS-CoV-2 proteins and human host proteins associated with immune, inflammatory, and host-response processes. This filtering step was based on biological relevance rather than on a fixed numerical centrality cut-off. The resulting focused subnetwork was subsequently integrated with the selected bacterial and host immune components. For the bacterial component, key *A. baumannii* (strain ATCC 17978) proteins involved in virulence, antibiotic resistance, and immune modulation were selected based on literature evidence and STRING database annotations (Szklarczyk et al., 2023), using a confidence cutoff of 0.7 to ensure high-quality interactions.

All interaction datasets were imported into Cytoscape v3.10.0 (Kohl et al., 2011) Network merging was conducted using the “Merge Networks” function, aligning nodes based on their shared gene or protein names. In cases where relationships between nodes were not automatically established but were supported by biological evidence, manual curation was performed. These manually curated edges were used to represent literature-supported functional associations across viral, bacterial, and host components and should not necessarily be interpreted as direct physical protein–protein interactions. The curated associations were rechecked against the supporting literature to verify their biological relevance.

For example, a functional association between the bacterial LpxC pathway and human TLR4 was incorporated based on the role of LpxC in lipid A/LPS biosynthesis and the experimentally demonstrated TLR4-dependent recognition of *A. baumannii* LPS (García-Quintanilla et al., 2016). Similarly, the *A. baumannii* outer membrane protein OmpA was linked to TLR2 based on experimental evidence supporting OmpA-mediated activation of TLR2-dependent inflammatory signaling (Skerniškytė et al., 2021; Wielento et al., 2022; Zhang et al., 2022). On the viral side, ORF3a was linked to NF-κB based on evidence of ORF3a-mediated activation of pro-inflammatory signaling (Nie et al.), while the Spike protein was linked to ACE2 to represent the well-established receptor-mediated mechanism of SARS-CoV-2 entry into host cells (Wrobel, 2023).

The tripartite model was designed to represent the molecular interplay among three biological components: SARS-CoV-2, *A. baumannii*, and the human host. The viral–host component captured interactions between SARS-CoV-2 proteins and human proteins, whereas the bacterial component represented *A. baumannii* proteins potentially involved in host-related pathogenic processes. These interaction layers were integrated through shared or functionally relevant human immune proteins to generate a unified host–virus–bacterium network. This integrated model enabled simultaneous examination of the three components and facilitated the identification of hub proteins and host immune pathways potentially involved in the inflammatory response associated with co-infection.

### Network analysis and visualization

Topological analysis of the integrated protein interaction network was conducted using the CytoHubba plugin in Cytoscape v3.10.0. To assess the robustness of hub identification, network centrality analysis was performed using six cytoHubba algorithms: Maximal Clique Centrality (MCC), Degree, Maximum Neighborhood Component (MNC), Bottleneck, Density of Maximum Neighborhood Component (DMNC), and Closeness. The top 30 ranked nodes obtained from each algorithm were compared, and their recurrence across the different centrality measures was evaluated to identify consistently prioritized nodes.

The 30 highest-ranked proteins according to the MCC algorithm were retained as the principal hub set presented in the main network analysis, while the other five centrality measures were used to assess the robustness and recurrence of these prioritized proteins.

### Functional enrichment analysis

To investigate the biological roles of key human immune proteins within the network, functional enrichment analysis was performed on the top 10 human proteins (as ranked by MCC) using two complementary tools:

- DAVID (Database for Annotation, Visualization, and Integrated Discovery) v2026, which enabled Gene Ontology (GO) annotation and pathway mapping for *Biological Processes*, *Molecular Functions*, and *Cellular Components.* The official DAVID Gene List format was used, and *Homo sapiens* was selected as the background population.
- ShinyGO v0.76, an interactive web-based platform for enrichment analysis, was also employed to visualize enriched GO terms and associated gene networks. *FDR-corrected p-values < 0.05* were considered statistically significant. The tool provided additional insights through tree plots and bar charts.

This dual-tool strategy allowed complementary comparison of the enrichment results and provided additional support for the functional interpretation of the selected human proteins. Only human immune-related proteins were included in this enrichment phase, while viral and bacterial proteins were excluded to avoid background bias.

### Ligand dataset preparation and compound collection

An initial chemical library was constructed to target TLR4 based on five well-documented natural compounds with reported inhibitory activity: curcumin (cid969516) (Wu et al., 2024), resveratrol (cid445154) (Zhao et al., 2011), parthenolide (cid72251185) (Molteni et al., 2018), Baicalin (cid64982) (Luo et al., 2024), and Chrysin (cid5281607) (Molteni et al., 2018). These compounds were used as reference scaffolds to retrieve structurally similar analogs from the PubChem database (Zavodszky et al., 2009) (https://pubchem.ncbi.nlm.nih.gov/). Preliminary comparison using a lower similarity threshold of 80% retrieved approximately 10,000 compounds. Therefore, the 90% threshold was retained to obtain a more focused chemical space enriched in close structural analogs of the selected natural-product scaffolds (Lipinski, 2004).

Using the PubChem Structure Similarity Search tool, compounds sharing ≥90% structural similarity with each reference ligand were identified. The initial compound collection comprised 5,391 structures. After application of the predefined filtering procedure, including Lipinski’s Rule of Five (Ohto et al., 2012), together with duplicate removal, 2,820 compounds were retained for subsequent molecular docking.

Following similarity-based filtering and duplicate removal, the retained compounds were downloaded in SDF format and further processed using LigPrep Schrödinger Suite, (Schrodinger2025-3, LLC, New York, NY, United States of America) for 3D structure optimization, tautomer generation, stereoisomer enumeration, and protonation state adjustment at physiological pH (7.0 ± 0.5).

### Protein preparation and molecular docking

TLR4 was selected as the primary docking target based on its network prioritization, its established role in LPS-mediated innate immune and inflammatory signaling (Resman et al., 2008), and the availability of a structurally characterized TLR4/MD-2 ligand-binding cavity suitable for structure-based screening (Cuadrado et al., 2018; Fu et al., 2024). The crystallographic structure of the human TLR4/MD-2 complex (PDB ID: 4G8A) was obtained from the Protein Data Bank (Banerjee et al., 2024). To specifically target the MD2 subunit, which is critical for ligand recognition and lipopolysaccharide (LPS) binding, only chains B and D (corresponding to MD2 and TLR4, respectively) were retained, while chains A and C were excluded. Protein preparation was performed using the Protein Preparation Wizard in Schrödinger Maestro. This included assignment of bond orders, addition of hydrogen atoms, optimization of hydrogen bonding networks, protonation state prediction at physiological pH (7.0 ± 0.5), and restrained energy minimization using the OPLS4 force field.

The active site was defined using Receptor Grid Generation centered around the native ligand binding pocket. The final grid box encompassed the coordinates X = −14.925, Y = −19.741, Z = −10.930. This site contains key amino acid residues involved in ligand binding, notably PHE151, ILE63, and PHE76, which are known to engage in hydrophobic and π–π stacking interactions with small molecules (Jorgensen et al., 1983; Lu et al., 2021).

Molecular docking was carried out using the Glide XP (Extra Precision) mode. The prepared ligand library was screened against the defined MD2 pocket, and docking scores, interaction types, and binding conformations were analyzed.

To further characterize the binding interactions, Discovery Studio Visualizer was used to examine hydrogen bonds, hydrophobic contacts, and π–interactions within each protein–ligand complex.

### ADMET profiling and biological activity prediction

The top-ranking ligands from molecular docking were subjected to a comprehensive *in silico* ADMET evaluation to assess their drug-likeness, pharmacokinetic behavior, and toxicity risks. The ADMETlab 3.0 platform (Decherchi and Cavalli, 2020) was used to predict key parameters, including oral bioavailability (OB%), Caco-2 permeability, human intestinal absorption (HIA), blood-brain barrier (BBB) penetration, cytochrome P450 (CYP450) inhibition potential (notably CYP3A4 and CYP2D6), plasma protein binding (PPB), and elimination half-life (T1/2).

Toxicological risks were further evaluated using the ProTox-III server (Ouassaf et al., 2025), which provides predictions for acute oral toxicity (LD_50_), toxicity class, and potential hepatotoxic, carcinogenic, immunotoxic, mutagenic, and cytotoxic effects.

### Molecular dynamics simulations

To further assess the stability and dynamic behavior of the most promising protein-ligand complexes, molecular dynamics (MD) simulations were performed using the Desmond v3.6 module integrated within Schrödinger Maestro. Complexes were selected based on their top docking scores and favorable ADMET profiles.

Each protein-ligand complex was embedded in an orthorhombic water box using the TIP3P water model, with a 10 Å buffer distance from the protein surface (Kim et al., 2023). The system was neutralized by adding appropriate counterions (Na^+^ or Cl^−^), and 0.15 M NaCl was added to simulate physiological ionic strength. OPLS4 force field was employed for all molecular parameterizations (Balic et al., 2020).

The system underwent a default relaxation protocol, including: i. Energy minimization to relieve any steric clashes, ii. Equilibration steps using restrained and unrestrained NVT and NPT ensembles. The production run was carried out for 100 nanoseconds under isothermal-isobaric (NPT) conditions at 300 K and 1.01325 bar using the Martyna–Tobias–Klein barostat and Nosé–Hoover thermostat. The RESPA integrator was used with a time step of 2.0 fs for bonded and near interactions, and 6.0 fs for far interactions (Hedayati et al., 2025).

Trajectory data were recorded every 100 ps, and analyses were conducted using the Simulation Interaction Diagram (SID) tool. Key parameters such as Root Mean Square Deviation (RMSD), Root Mean Square Fluctuation (RMSF), hydrogen bond stability, and protein-ligand contacts were examined to evaluate complex stability and interaction persistence (Baumgarten et al., 2006).

## Results

### Network-based integration of host–virus–bacteria interactions

An integrated protein–protein interaction (PPI) network representing SARS-CoV-2, *A. baumannii*, and the human host was generated to characterize the molecular landscape of the co-infection. The bacterial component included proteins representing major functional categories associated with *A. baumannii* pathogenicity, including LPS biosynthesis, outer membrane structure, biofilm formation and adhesion, multidrug efflux, and environmental sensing (Table 1).

**TABLE 1:** Selected *Acinetobacter baumannii* proteins and their functional roles.

Category	Proteins
LPS biosynthesis enzymes	LpxA, LpxB, LpxC, KdsA, KdsB
Outer membrane proteins	CarO, OmpA, Ata
Biofilm and adhesion factors	fimA, Bap
Multidrug efflux components	AdeA, AdeB
Environmental sensors	KdpB, KdpD

The host component included immune-related proteins involved in pathogen recognition, inflammatory signaling, antiviral responses, and viral entry, including TLR2, TLR4, IL6, IL8, TNF, ACE2, NF-κB, STAT3, and TBK1 (Table 2). Integration of these host proteins with the viral–host and bacterial interaction components provided the basis for the tripartite host–virus–bacterium network.

**TABLE 2:** Human immune-related proteins included in the model.

Protein	Function	Reference
TLR2	Bacterial pattern recognition	García-Quintanilla et al. (2016)
TLR4	LPS recognition	Molteni et al. (2016), García-Quintanilla et al. (2016)
IL6	Inflammatory cytokine	Molteni et al. (2016)
IL8	Neutrophil recruitment	Molteni et al. (2016)
TNF	Proinflammatory mediator	Molteni et al. (2016)
ACE2	Viral entry receptor	Nie et al. (2023)
NF-κB	Transcription factor, inflammation	Molteni et al. (2016)
STAT3	Cytokine signaling	Niu et al. (2018)
TBK1	IRF3 activation, antiviral response	Molteni et al. (2016)

The original viral–host interaction network comprised 7,672 nodes and 51,853 edges. To reduce network complexity while retaining components relevant to the co-infection context, the network was biologically filtered by retaining SARS-CoV-2 proteins and human host proteins associated with immune, inflammatory, and host-response processes. This filtering was based on biological relevance rather than a fixed numerical centrality cutoff. Following integration with the selected bacterial and host immune components, the resulting network comprised 218 nodes and 4,713 edges (Supplementary Figure S1).

To further evaluate the robustness of hub identification, the top 30 ranked nodes obtained independently from each of the six centrality algorithms were compared (Supplementary Table S1). Because the six Top-30 lists were not identical, their union comprised 37 unique nodes, which are reported in Supplementary Table S1 together with their recurrence across the different centrality metrics The final subnetwork composed of the top 30 proteins ranked according to the MCC metric is visualized in Figure 1 and Table 3 **,** revealing a dense and highly interconnected structure. The network highlights critical viral proteins (e.g., NSP3, NSP12, ORF3b), key immune factors (e.g., TLR4, IL6), and central bacterial effectors (e.g., LpxC, KdsA, Bap), suggesting that these components may collectively orchestrate pro-inflammatory responses, immune evasion, and enhanced pathogenesis in co-infection scenarios.

**FIGURE 1:**
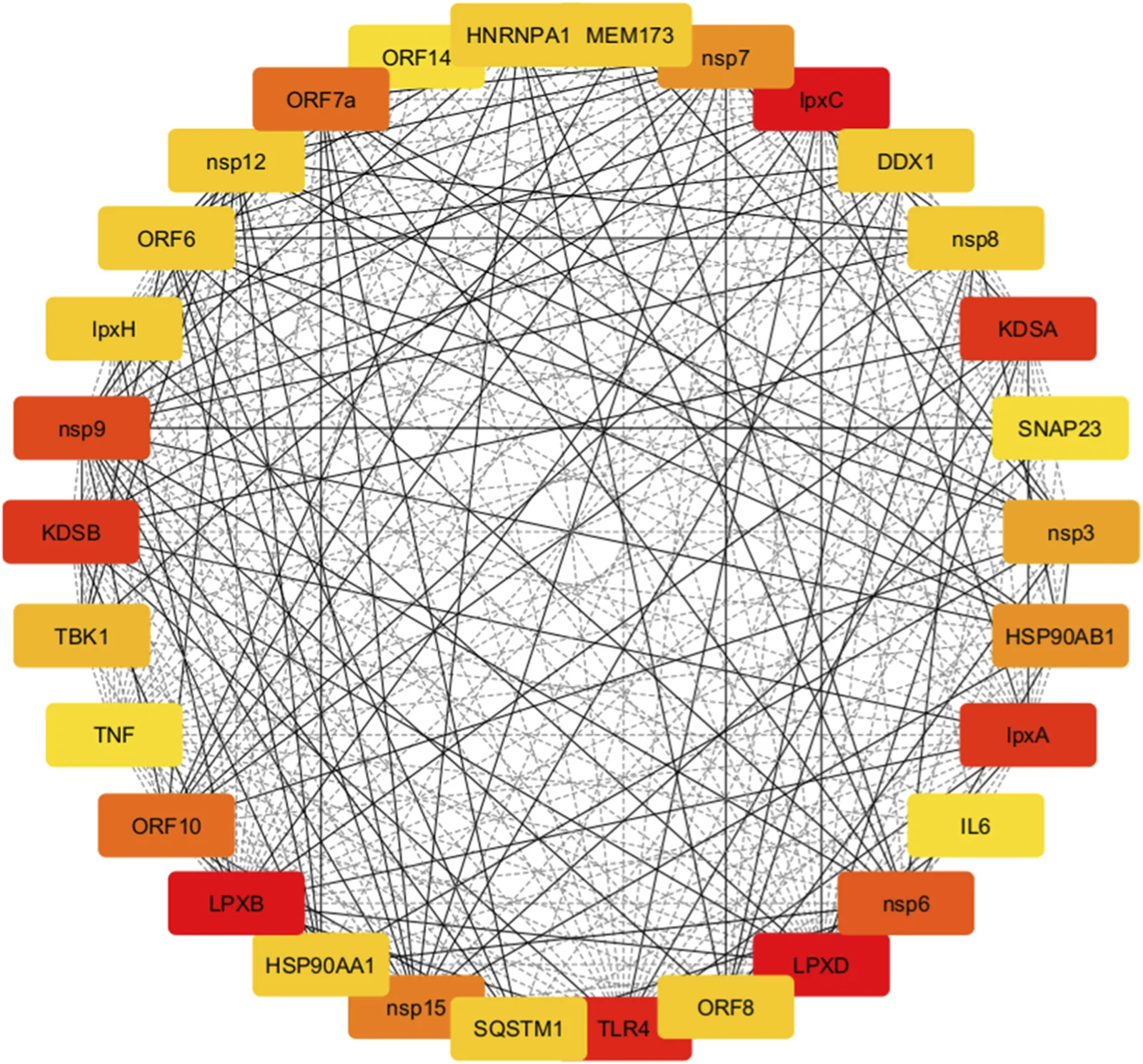
Integrated synergistic biological network of SARS-CoV-2, *Acinetobacter baumannii*, and human immune proteins. This network was built by merging protein–protein interaction data from multiple sources using Cytoscape. Proteins were categorized into three classes (viral, bacterial, immune). The network visualizes cross-domain interactions, highlighting convergence points such as TLR4, NF-κB, and LpxC as potential synergistic hubs.

**TABLE 3:** Classification of viral, bacterial, and host proteins involved in the tripartite interaction network with associated scores.

Gene/Protein	Score	Category	Gene/Protein	Score	Category
lpxC	726.0	Bacterial	HSP90B1	24.0	Host immune
LPXD	726.0	Bacterial	ARPC3	24.0	Host immune
LPXB	726.0	Bacterial	nsp6	20.0	Viral
TLR4	722.0	Host immune	ORF8	16.0	Viral
lpxA	720.0	Bacterial	ORF10	12.0	Viral
KDSB	720.0	Bacterial	ORF7a	12.0	Viral
KDSA	720.0	Bacterial	nsp3	9.0	Viral
nsp9	60.0	Viral	lpxH	6.0	Bacterial
nsp15	53.0	Viral	HSP90AB1	6.0	Host immune
nsp7	42.0	Viral	DDX1	6.0	Host immune
ORF3b	31.0	Viral	LARP1	6.0	Host (Non-immune)
nsp8	30.0	Viral	TMEM173	6.0	Host immune
nsp13	26.0	Viral	SQSTM1	6.0	Host immune
nsp10	24.0	Viral	HNRNPA1	6.0	Host (Non-immune)
nsp12	24.0	Viral	IL6	4.0	Host immune

### Functional enrichment analysis of the top 10 human proteins

To gain insights into the functional roles of the top-ranked human proteins identified in our virus–bacteria–host PPI network, we performed Gene Ontology (GO) and KEGG pathway enrichment analyses using both DAVID and ShinyGO tools, focusing only on the top 10 human proteins extracted from the MCC ranking.

#### GO enrichment using DAVID

The GO enrichment analysis revealed functional associations across the three main GO categories: Biological Process (BP), Cellular Component (CC), and Molecular Function (MF) (Figure 2). Within BP, the selected terms highlighted processes related to antiviral and inflammatory responses, including defense response to virus, innate immune response, inflammatory cytokine production, interferon-beta production, response to Gram-negative bacteria, response to lipopolysaccharide, IL-6 production, and TNF production.

**FIGURE 2:**
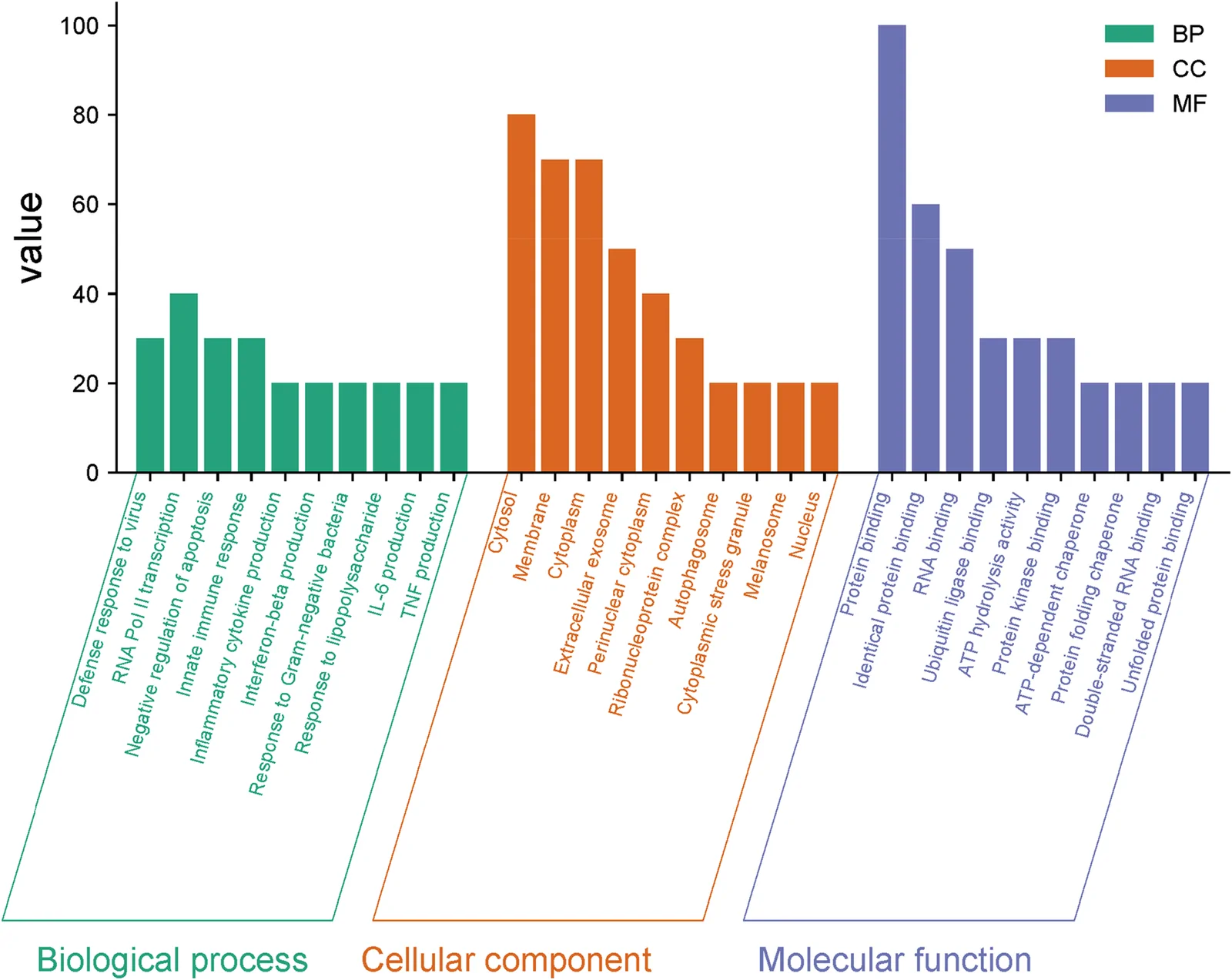
Gene ontology (GO) enrichment profile of the selected human proteins using DAVID 2026. The bar plot shows the top 10 terms for biological process (BP), cellular component (CC), and molecular function (MF). Bar values represent the percentage of the analyzed proteins associated with each GO term.

In the CC category, the selected proteins were predominantly associated with the cytosol, membrane, cytoplasm, extracellular exosome, and perinuclear region of the cytoplasm, together with ribonucleoprotein complexes, autophagosomes, cytoplasmic stress granules, melanosomes, and the nucleus.

Regarding MF, protein binding represented the most frequently associated function, followed by identical protein binding and RNA binding. Additional terms included ubiquitin ligase binding, ATP hydrolysis activity, protein kinase binding, ATP-dependent chaperone activity, protein-folding chaperone activity, double-stranded RNA binding, and unfolded protein binding.

DAVID analysis also identified significantly enriched KEGG pathways (FDR < 0.05), including *Salmonella* infection, NOD-like receptor signaling, lipid and atherosclerosis, Shigellosis, PI3K-Akt signaling, IL-17 signaling, *Yersinia* infection, and fluid shear stress and atherosclerosis (Figure 3). Among these pathways, IL-17 signaling showed the highest fold enrichment (37.40), while several infection- and inflammation-related pathways showed recurrent involvement of TLR4, IL6, and HSP90 family proteins. These findings further highlight the enrichment of host immune and inflammatory signaling processes within the analyzed protein set. The complete DAVID KEGG enrichment results, including gene counts, fold enrichment, P-values, FDR values, and associated genes, are provided in Supplementary Table S2.

**FIGURE 3:**
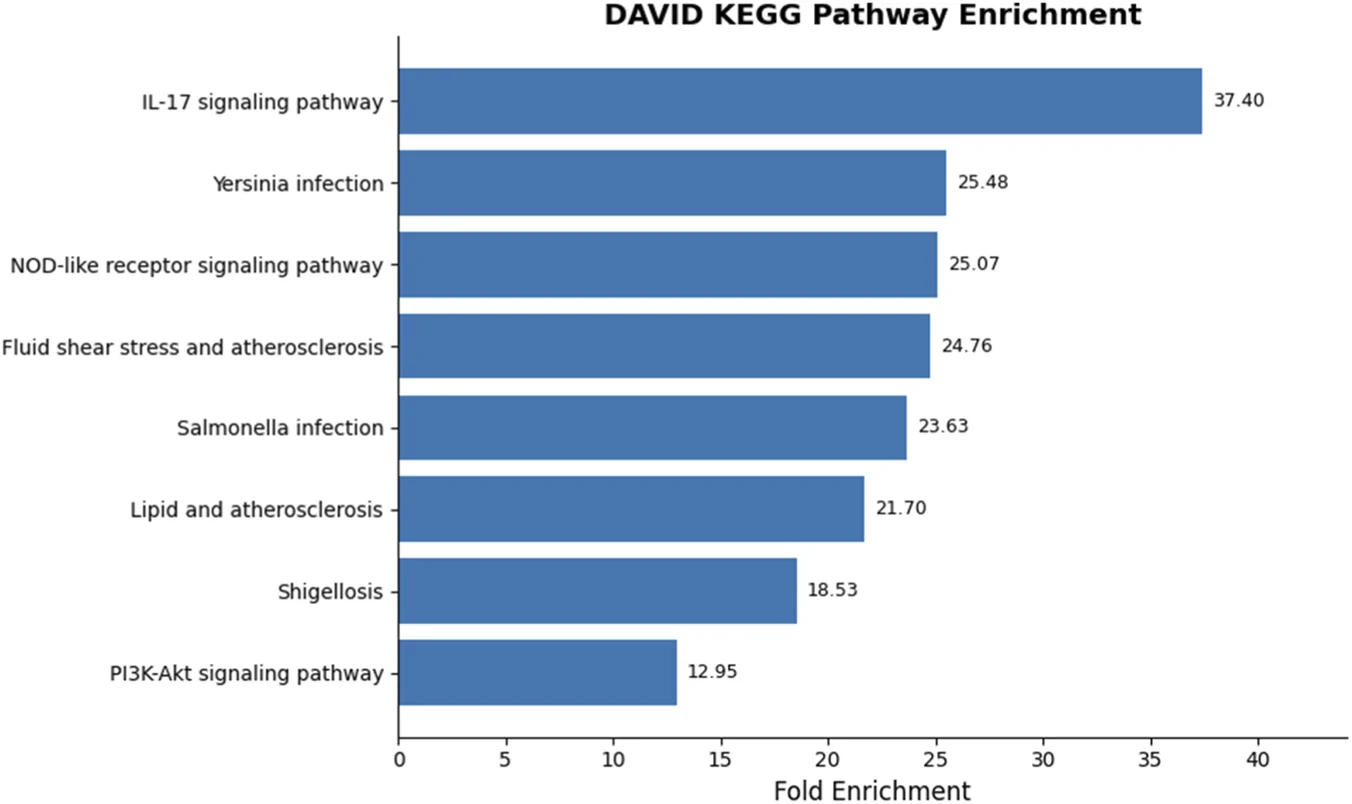
DAVID KEGG pathway enrichment analysis of the selected human proteins. Bars represent the fold enrichment of significantly enriched KEGG pathways (FDR < 0.05).

#### Pathway enrichment using ShinyGO

Complementary analysis with ShinyGO confirmed and extended these findings by mapping the top 10 human proteins to highly enriched KEGG pathways. The barplot in Figure 4A shows that several immune-related and infection-associated pathways were enriched, including the IL-17 signaling pathway, *Salmonella* infection, Toll-like receptor signaling, and NOD-like receptor signaling pathways. The presence of cancer-related pathways such as prostate cancer and PI3K-Akt signaling further highlights the potential role of these proteins in the inflammation-to-cancer transition, a process commonly associated with chronic infection (Feng et al., 2024).

**FIGURE 4:**
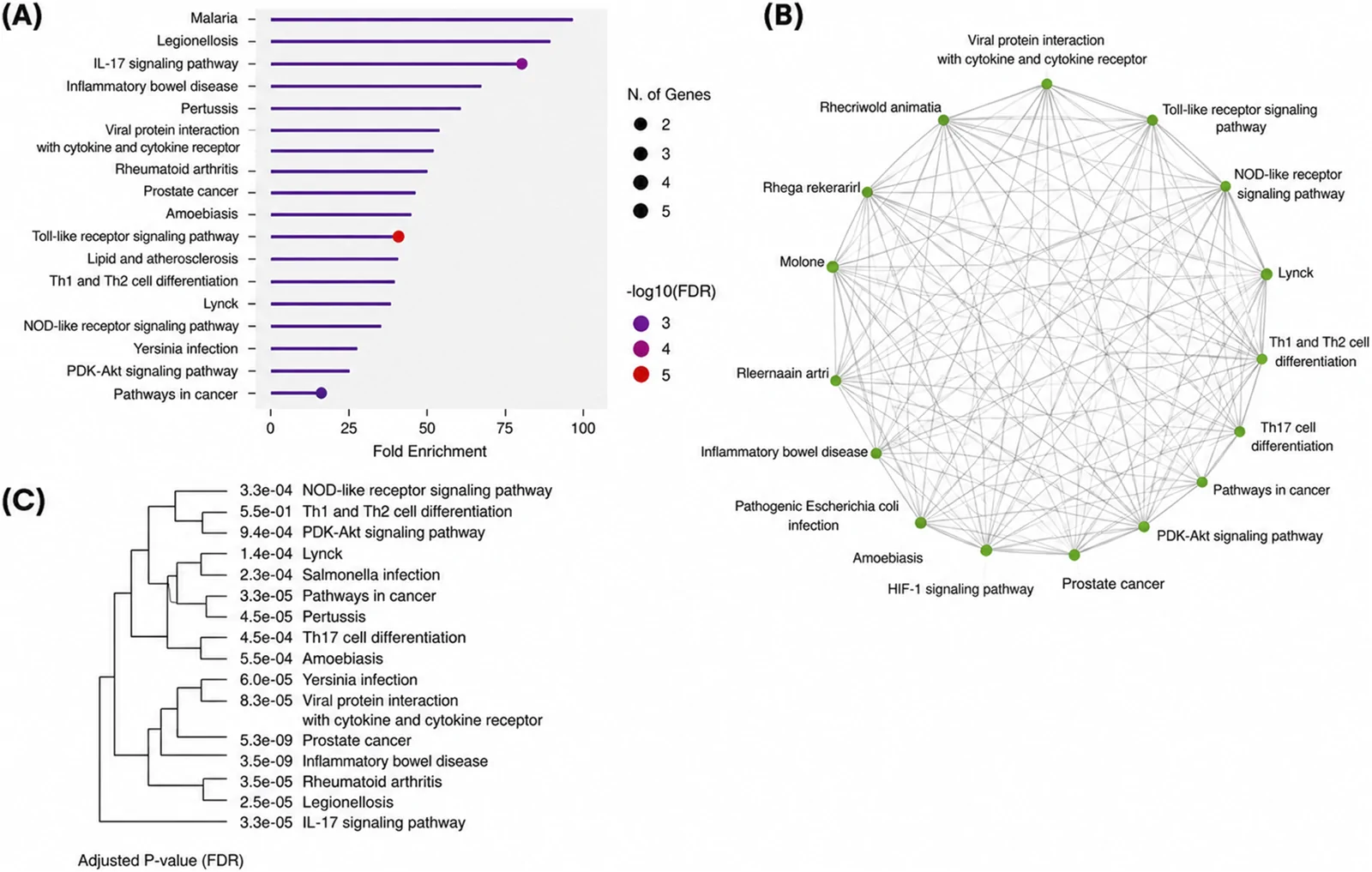
KEGG pathway analysis of top human proteins using ShinyGO: **(A)** enrichment barplot, **(B)** functional connectivity network, and **(C)** hierarchical clustering of significant pathways.

The pathway connectivity network shows dense interlinkages among these enriched pathways, with key hubs such as *Salmonella* infection and PI3K-Akt signaling demonstrating high centrality (Figure 4B). This suggests that these pathways serve as convergence points for multiple immune and stress signals.

The hierarchical clustering of pathways further confirms the existence of functionally related modules, where inflammation, immune response, and cancer pathways cluster together, reinforcing the central hypothesis of our study: that human proteins interacting with both viral and bacterial components may serve as pivotal nodes in immune regulation and disease progression (Figure 4C).

As shown in Table 4, the most significantly enriched KEGG pathways include multiple immune and infection-related processes, with IL6 and TLR4 appearing recurrently across nearly all top pathways. Their frequent co-occurrence underscores their central role in host immune regulation during infection. Notably, pathways such as Malaria, Legionellosis, and IL-17 signaling exhibited the highest fold enrichment, while others like prostate cancer, PI3K-Akt signaling, and NOD-like receptor signaling support the idea that chronic immune activation may bridge infectious stimuli to oncogenic progression. These enrichments further validate the relevance of the selected human targets in mediating both pathogen recognition and inflammation-driven disease outcomes.

**TABLE 4:** Most significantly enriched KEGG pathways for selected human target proteins.

Fold enrichment	Pathway	Genes
103.769	Path:hsa05144 Malaria	IL6 TLR4
89.205	Path:hsa05134 Legionellosis	IL6 TLR4
82.011	Path:hsa04657 IL-17 signaling pathway	HSP90AB1 IL6 HSP90B1
78.226	Path:hsa05321 Inflammatory bowel disease	IL6 TLR4
66.904	Path:hsa05133 Pertussis	IL6 TLR4
55.672	Path:hsa05135 *Yersinia* infection	ARPC3 IL6 TLR4
55.268	Path:hsa05323 Rheumatoid arthritis	IL6 TLR4
52.419	Path:hsa05215 Prostate cancer	HSP90AB1 HSP90B1
51.051	Path:hsa05132 *Salmonella* infection	ARPC3 HSP90AB1 IL6 TLR4 HSP90B1
50.343	Path:hsa05142 Chagas disease	IL6 TLR4
49.85	Path:hsa05146 Amoebiasis	IL6 TLR4
49.366	Path:hsa04620 Toll-like receptor signaling pathway	IL6 TLR4
47.52	Path:hsa05417 Lipid and atherosclerosis	HSP90AB1 IL6 TLR4 HSP90B1
47.08	Path:hsa04659 Th17 cell differentiation	HSP90AB1 IL6
46.648	Path:hsa04066 HIF-1 signaling pathway	IL6 TLR4
41.678	Path:hsa04621 NOD-like receptor signaling pathway	HSP90AB1 IL6 TLR4
38.716	Path:hsa05130 Pathogenic *Escherichia coli* infection	ARPC3 IL6 TLR4
36.845	Path:hsa04915 Estrogen signaling pathway	HSP90AB1 HSP90B1
28.727	Path:hsa04151 PI3K-Akt signaling pathway	HSP90AB1 IL6 TLR4 HSP90B1
14.391	Path:hsa05200 Pathways in cancer	HSP90AB1 IL6 HSP90B1

### Molecular docking study of selected compounds targeting TLR4

Based on the preceding network pharmacology and enrichment analyses, TLR4 emerged among the prioritized human proteins associated with immune and inflammatory pathways relevant to viral and bacterial infection. Although TLR4 was identified in two of the six centrality-based Top-30 lists, its network prioritization together with its recurrent involvement in enriched GO terms and KEGG pathways supported its consideration for subsequent structure-based investigation. Its final selection was therefore based on combined network, functional enrichment, biological, and structural considerations rather than centrality ranking alone. A preliminary similarity search at the 80% threshold retrieved approximately 10,000 compounds. Applying the more stringent 90% similarity criterion, followed by duplicate removal and the predefined filtering procedure. A curated dataset of 2,820 compounds were generated, all sharing ≥90% structural similarity with five known TLR4-modulating natural products: Chrysin derivatives, curcumin, Baicalin (*Scutellaria baicalensis*), resveratrol and parthenolide. All compounds were filtered according to Lipinski’s Rule of Five to ensure drug-likeness. Docking simulations were carried out using the human TLR4/MD-2 complex (PDB ID: 4G8A), focusing on the MD-2 binding cavity where the known TLR4 antagonist Eritoran binds. Figure 5 illustrates the TLR4/MD-2 complex and the binding mode of Eritoran. The binding cavity lies deep within MD-2 and is characterized by nonpolar and aromatic residues (e.g., PHE76, PHE104, ILE94, LEU61, VAL135), which promote hydrophobic and π–π stacking interactions, while polar residues such as SER120 and ARG264 assist in ligand anchoring via hydrogen bonding. Eritoran establishes a combination of hydrogen bonds and hydrophobic interactions, forming a benchmark profile for evaluating candidate ligands. To gain deeper insights into the physicochemical environment of the binding pocket, surface mapping was performed (Figure 6), revealing that the MD-2 cavity is dominantly hydrophobic with selective polar entry zones. The electrostatic potential is mostly neutral to slightly negative, favoring lipophilic or amphipathic ligands. Among the 2,820 screened compounds, three candidates emerged with superior docking scores compared to the reference antagonist Eritoran (−9.10 kcal/mol): CID5898023 (curcumin-derived, −10.46 kcal/mol), CID5403474 (Chrysin-derived, −10.02 kcal/mol), and CID74977829 (Baicalin–derived, −9.58 kcal/mol). These results indicate favorable predicted docking interactions within the TLR4/MD-2 binding cavity and supported the prioritization of these compounds for subsequent ADMET and molecular dynamics analyses (Table 5; Figure 7).

**FIGURE 5:**
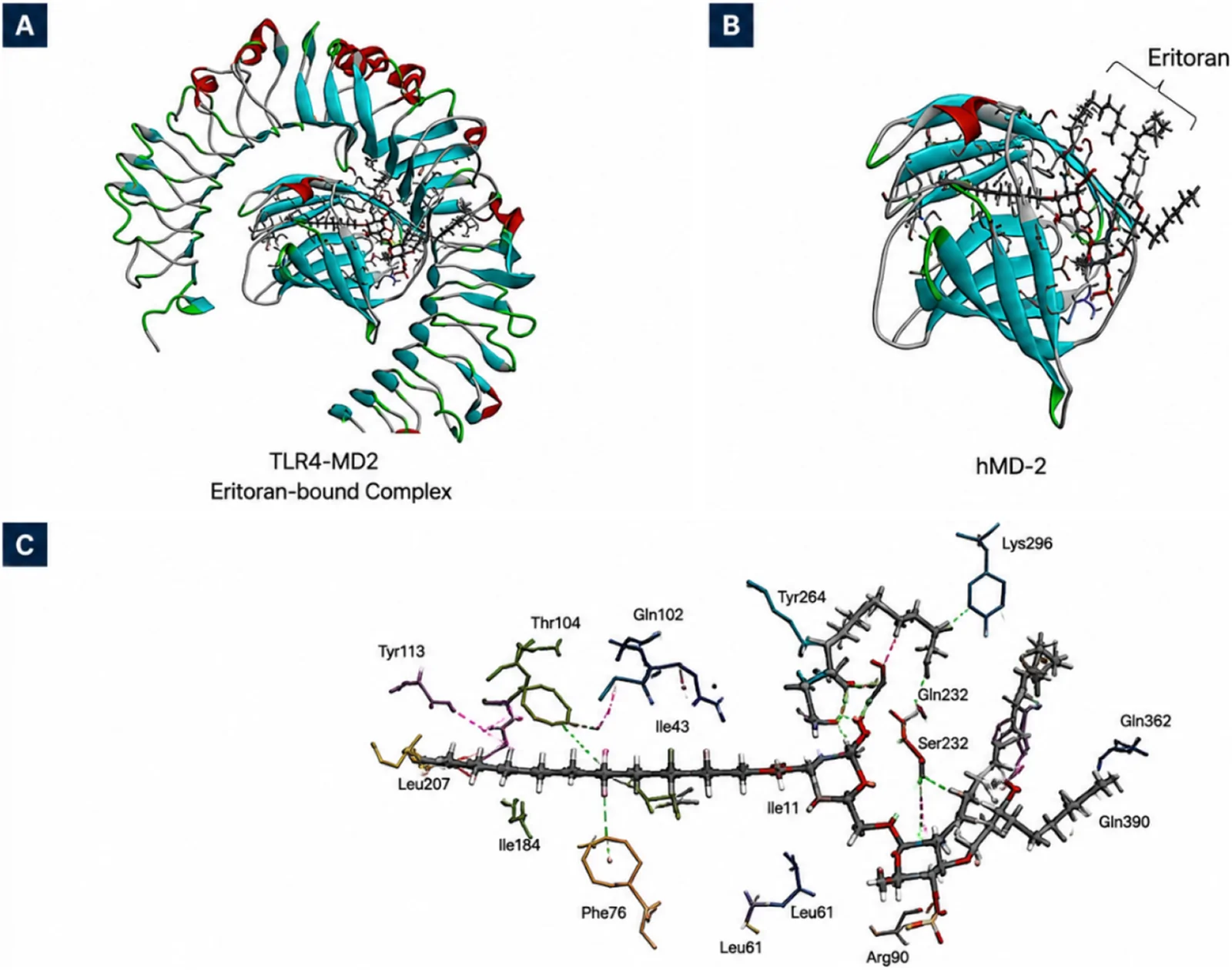
Structural representation of the TLR4/MD-2 complex and binding conformation of the reference antagonist Eritoran. **(A)** Full 3D structure of the human TLR4/MD-2 complex (PDB ID: 4G8A) highlighting the ligand-binding cavity at the interface between TLR4 and its co-receptor MD-2. **(B)** Close-up view of human MD-2 (hMD-2) with the antagonist Eritoran bound inside the hydrophobic pocket. Eritoran’s flexible aliphatic chains occupy the internal cavity mimicking the lipid A moiety of LPS. **(C)** Detailed 2D interaction map of Eritoran with key amino acid residues forming the MD-2 binding pocket. Notable residues involved in hydrophobic (LEU61, ILE94, PHE76) and hydrogen bonding (SER120, GLU92) interactions are visualized, confirming strong mimicry of endotoxin binding.

**FIGURE 6:**
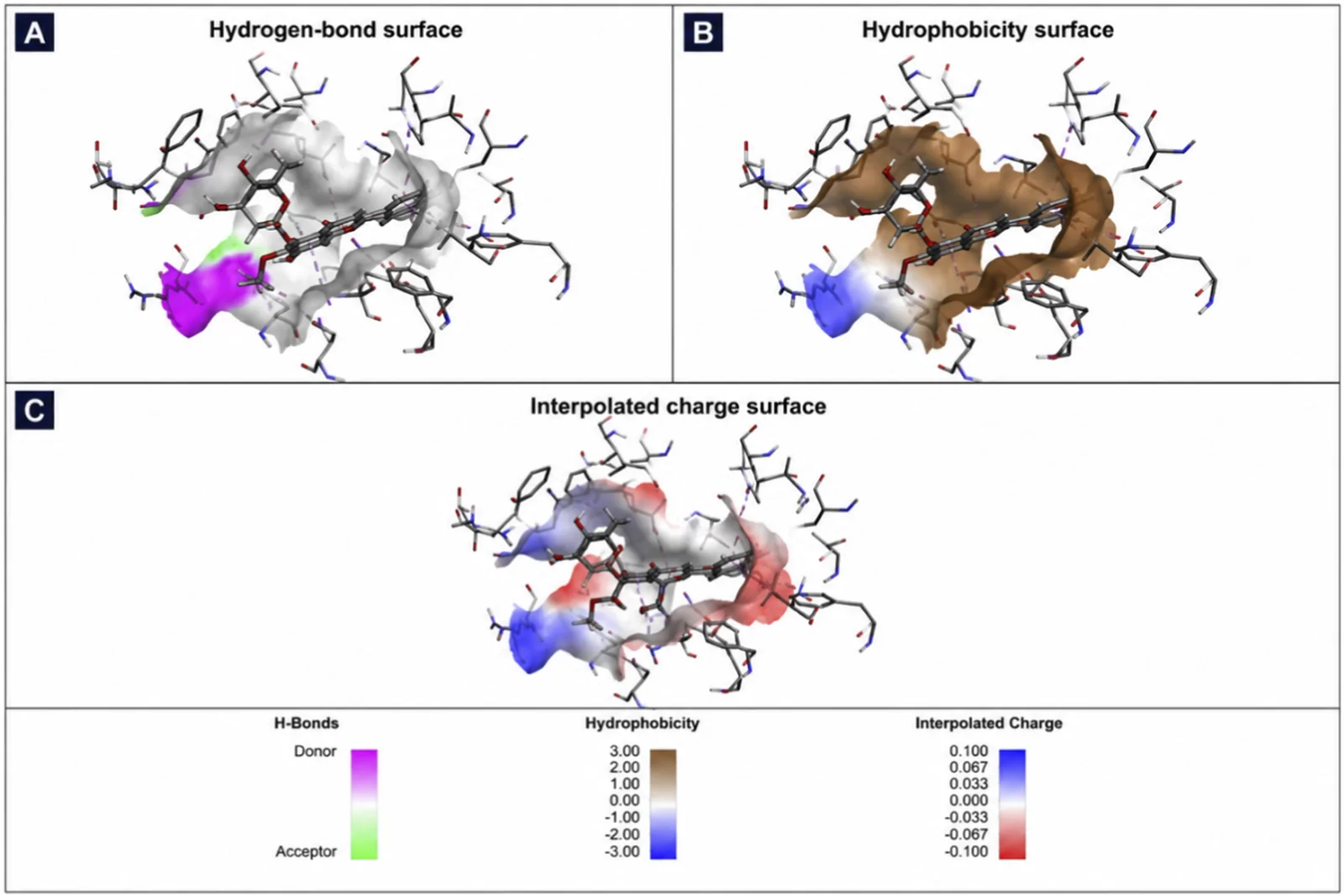
Surface property analysis of the MD-2 binding pocket within the TLR4/MD-2 complex (PDB ID: 4G8A) highlighting key physicochemical features relevant to ligand docking. From left to right: **(A)** Hydrogen bond donor (magenta) and acceptor (green) regions; **(B)** Hydrophobicity map showing a predominantly nonpolar inner cavity (brown) with a hydrophilic entrance (blue); **(C)** Electrostatic surface potential ranging from mildly negative (red) to neutral and slightly positive zones (blue), supporting the preference for amphipathic ligand.

**TABLE 5:** Summary of interactions between top compounds and 4G8A.

PubChem ID	Residue name	Type	Category	Interval distance (A°)
CID74977829 S _XP=_-9.585 kcal/mol	Phe 147-phe76	Pi-pi	hydrophobic	4.2–5.46
	Ile94	alkyl		
	Tyr102 phe119 leu61 ile61 ile46 val35	Pi-alkyl		
CID5403474 S _XP=_-10.021 kcal/mol	Ile46 ile63	Pi-sigma	hydrophobic	3.9–5.28
	Phe76 phe104	Pi-pi		
	Ile94 val135 Ile117 Leu61	Pi-alkyl		
CID5898023 S _XP=_-10.46 kcal/mol	Phe151	hydrophobic	hydrophobic	3.7–5.7
	Val135 ile44 ile46 ile63 ile117 leu17 leu61 cys133	Alkyl		
	Phe76 phe104 phe151 ile94 ile117 leu78 cys133 val135	Pi-alkyl		
	Tyr102	Conv-h-bond	h-bond	2.31

**FIGURE 7:**
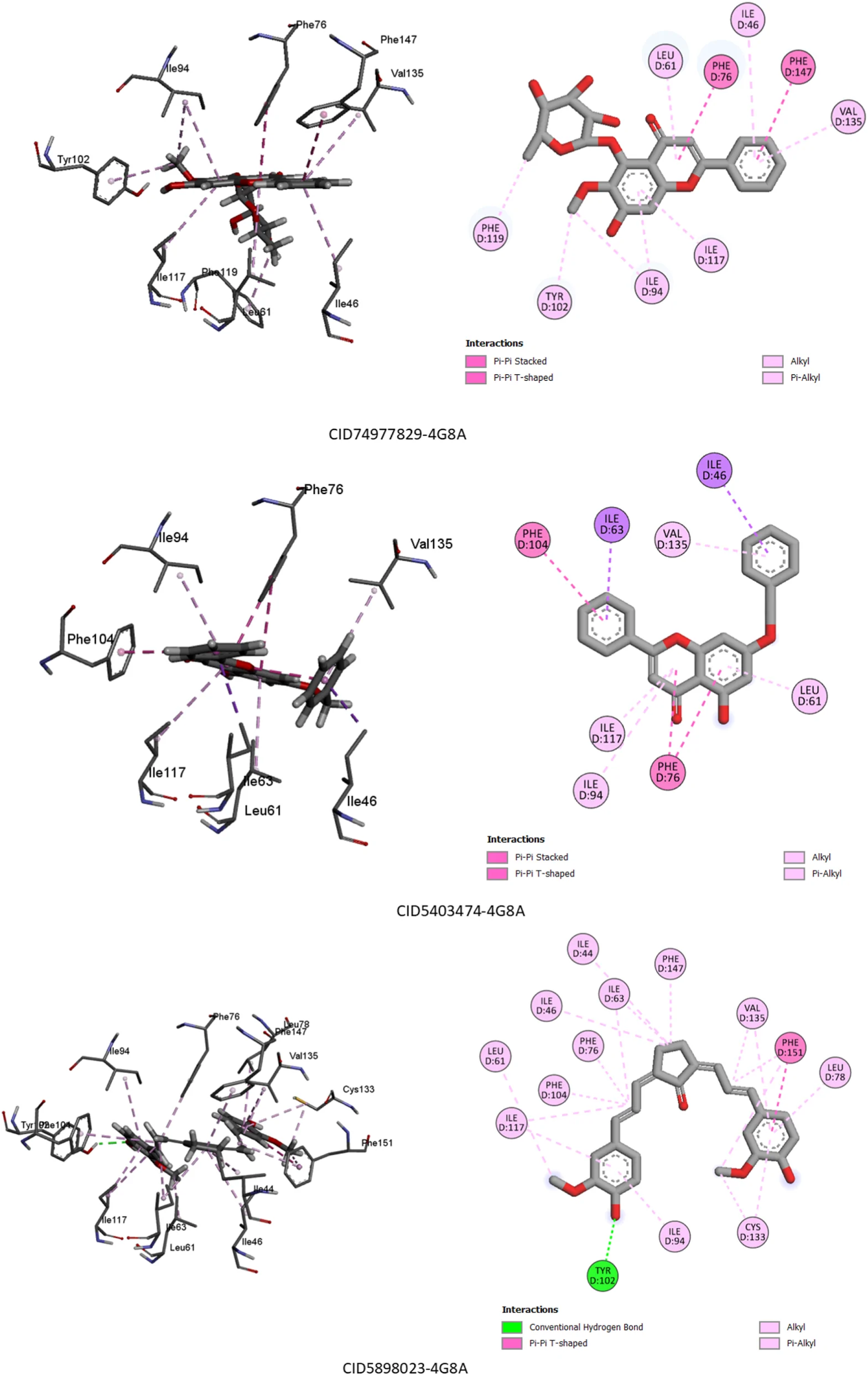
Comparative 2D and 3D interaction diagrams of the top three natural ligands docked into the TLR4/MD-2 active site (PDB ID: 4G8A). The figure illustrates both the spatial conformation (3D) and detailed interaction profiles (2D) of CID5898023 (curcumin-derived), CID5403474 (Chrysin-derived), and CID74977829 (baicalin-derived) within the MD-2 binding cavity. Key interactions include hydrophobic contacts, π–π stacking, and hydrogen bonds with residues such as PHE76, ILE94, LEU61, SER120, and TYR102.

Among the three top-performing compounds, CID5898023 demonstrated the most favorable binding profile, with a strong conventional hydrogen bond at 2.31 Å involving TYR102, and multiple hydrophobic interactions including π–π stacking with PHE76, PHE104, and PHE151, and π–alkyl contacts with residues such as ILE94, ILE117, LEU61, and VAL135. Its dense interaction network, spanning both polar and nonpolar contacts, suggests a stable binding within the pocket. The presence of a phenolic scaffold reinforces its drug-likeness and supports further development.

CID5403474, with a binding score of −10.021 kcal/mol, exhibited significant π–π stacking with PHE76 and PHE104, and π–alkyl interactions with ILE94, VAL135, and ILE117. Although no hydrogen bonds were observed, its strong aromatic and alkyl contacts across the 3.9–5.28 Å range indicate a deep and stable insertion into the MD-2 binding cavity. This compound, structurally related to chrysin analogs, shows promis due to its compact, lipophilic nature.

Lastly, CID74977829 showed a binding score of −9.585 kcal/mol and established extensive hydrophobic interactions over a 4.2–5.46 Å interval. It engaged in π–π interactions with PHE147 and PHE76, along with alkyl and π–alkyl contacts involving ILE94, TYR102, PHE119, LEU61, and VAL135. While it lacked direct hydrogen bonding, its flexible aliphatic chains allowed excellent adaptation to the hydrophobic cavity, justifying its inclusion in further ADMET and molecular dynamics evaluations.

By comparison, the reference compound Eritoran achieved a docking score of −9.10 kcal/mol. It establishes multiple polar and hydrophobic interactions within the MD-2 pocket, yet its bulky, highly lipophilic nature may limit oral bioavailability and hinder downstream drug development.

In summary, all three natural compounds—CID5898023, CID5403474, and CID74977829—demonstrated higher binding affinities than the known antagonist, each exploiting different interaction mechanisms within the TLR4 binding pocket. These findings support their continued investigation through ADMET filtering and molecular dynamics simulations, particularly in the context of TLR4-targeted immunomodulation during viral–bacterial co-infections.

### In silico ADMET profiling of top TLR4-targeting natural compounds physicochemical properties

The molecular weights of the tested compounds range from 344.1 to 430.13 g/mol, all falling below the commonly accepted oral drug-likeness threshold of 500 g/mol (Table 6). In contrast, Eritoran displays a high molecular weight of 1312.84 g/mol, far exceeding the limit and indicating poor oral bioavailability potential.

**TABLE 6:** Physicochemical properties for the selected TLR4-targeting compounds.

Property	CID5403474	CID5898023	CID74977829	ERITORAN
Molecular weight	344.1	404.16	430.13	1312.84
TPSA	59.67	75.99	138.82	294
logP	4.809	3.563	1.142	2.093
logS	−4.072	−4.084	−2.978	−3.024
Flexibility	0.167	0.273	0.167	3.389

MW (g/mol)—molecular weight; should be <500 g/mol (Lipinski’s Rule). nRot—number of rotatable bonds; higher values indicate more flexibility. Flexibility—molecular flexibility; influences binding and permeability. TPSA(Å2)—topological polar surface area; should be <140 Å2 for good oral bioavailability.

Log S—aqueous solubility (log scale); lower values indicate poor solubility. Log P—partition coefficient (lipophilicity); should be <5 (Lipinski’s Rule).

Topological Polar Surface Area (TPSA) is another critical determinant of permeability and absorption. CID5403474 and CID5898023 have TPSA values of 59.67 Å^2^ and 75.99 Å^2^, respectively—well within the optimal range (<140 Å^2^). CID74977829, with a TPSA of 138.82 Å^2^, borders the upper limit, while Eritoran’s TPSA of 294 Å^2^ suggests extremely poor membrane permeability, consistent with its low human intestinal absorption (HIA).

The logP values of the compounds (4.809, 3.563, and 1.142) indicate that CID5403474 is highly lipophilic, which may enhance membrane permeability but could also increase non-specific binding. CID74977829 shows a more hydrophilic profile. Eritoran, with a logP of 2.093, shows moderate lipophilicity but is limited by its size and polarity.

Water solubility (logS) values reveal poor solubility across all candidates (logS < −2), with CID74977829 being the most soluble (−2.978) and CID5403474 the least (−4.072). Eritoran shows similar solubility issues (−3.024).

The flexibility score—an indicator of molecular rigidity—shows that the candidate molecules (scores ∼0.17–0.27) are significantly more rigid than Eritoran (3.389), which may favor better binding specificity.

### Drug-likeness and medicinal chemistry

Quantitative Estimate of Drug-likeness (QED) scores support CID5898023 (0.659) and CID5403474 (0.581) as the most promising candidates. Eritoran scores a poor 0.013, reflecting its unfavorable physicochemical profile (Table 7).

**TABLE 7:** Physicochemical properties of medicinal chemistry and drug-likeness properties for the selected TLR4-targeting compounds.

Property	CID5403474	CID74977829	CID5898023	ERITORAN
QED	0.581	0.483	0.659	0.013
SA Score	2	3	2	6
Lipinski Violations	0.0	0	0	1.0
Pfizer Rule	1	0	0	0
Golden Triangle	0	0	0	1.0

QED—quantitative estimate of drug-likeness; higher values indicate better drug-like properties.

SAscore—synthetic accessibility score; lower values indicate easier synthesis. Pfizer Rule—compliance with Pfizer’s drug-likeness criteria; accept/reject. Lipinski Rule—compliance with Lipinski’s Rule of Five; accept/reject. Golden Triangle—drug-likeness based on lipophilicity and MW; accept/reject.

Synthetic accessibility (SA Score) is optimal for CID5403474 and CID5898023 (score = 2), while Eritoran has a significantly more complex structure (SA = 6).

Lipinski’s Rule of Five is respected by all candidates, while Eritoran shows one violation, mainly due to its high molecular weight.

The Pfizer rule flags CID5403474 for potential toxicity, but all others comply. Similarly, only Eritoran fits the “Golden Triangle” filter, though this is not sufficient to offset its other limitations.

### Absorption

Caco-2 and MDCK cell models were used to predict intestinal absorption. The tested compounds (Table 8) show moderate-to-poor permeability, with CID5403474 performing slightly better (Caco-2: -4.885; MDCK: -4.683) compared to CID74977829 (Caco-2: -6.211). Eritoran shows comparable or worse values, suggesting that none of the molecules exhibit high passive permeability.

**TABLE 8:** ADME properties for the selected TLR4-targeting compounds (CID5898023, CID5403474, and CID74977829).

Property	CID5403474	CID5898023	CID74977829	ERITORAN
Absorption				
Caco-2	−4.885	−5.163	−6.211	−5.885
MDCK	−4.683	−4.847	−5.085	−5.168
Pgp-inhibitor	Yes	Yes	Non	Yes
HIA	100	100	75.25	0.039
F30%	0.655	0.975	0.781	0.725
Distribution				
PPB	98.482	97.638	87.82	97.19
VDss	0.196	−0.505	−0.274	0.102
BBB	0.281	0	0.069	0
Metabolism				
CYP1A2 inhibitor	Yes	Yes	Yes	Non
CYP2C9 inhibitor	Non	Yes	Non	Non
CYP3A4 inhibitor	Non	Non	Non	Non
Excretion				
CLplasma	4.445	7.595	2.84	2.254
T1/2	0.646	0.958	2.421	4.596

Caco-2 permeability estimates intestinal absorption, with more negative values indicating lower permeability. HIA% predicts human intestinal absorption, where values above 70% are high. P-gp inhibitor classification reflects interaction with P-glycoprotein. PPB indicates plasma protein binding, with values above 90% signifying strong binding and lower free drug levels. Vd (L/kg) represents drug distribution, where values above 1 L/kg suggest extensive tissue distribution. CYP inhibition values indicate the compounds’ potential to inhibit key cytochrome P450 enzymes, affecting drug metabolism. CL (clearance) represents the rate of drug elimination, with higher values indicating faster clearance. T1/2 (half-life) reflects the duration a drug remains in the body, where longer half-lives suggest prolonged effects.

However, P-glycoprotein inhibition differs starkly. CID5403474 and CID5898023 are predicted P-gp inhibitors (scores ∼0.99), which could enhance the intracellular retention of co-administered drugs. In contrast, CID74977829 shows almost no inhibition (0.006), and Eritoran is inactive.

Human Intestinal Absorption (HIA) is excellent for CID5403474 and CID5898023 (100%), moderate for CID74977829 (75.25%), and negligible for Eritoran (0.039%), consistent with its predicted poor oral absorption profile.

The predicted oral bioavailability at 30% (F30%) is highest for CID5898023 (0.975), suggesting favorable absorption and systemic exposure. Eritoran and the other compounds follow closely.

### Distribution

Plasma protein binding (PPB) is very high across all candidates (>87%), with CID5403474 showing the strongest binding (98.48%). Although high PPB may reduce free drug concentration, it also prolongs the circulation time.

The volume of distribution at steady state (VDss) ranges from −0.505 (CID5898023) to 0.196 (CID5403474), suggesting limited tissue distribution. Negative VDss values often imply confinement to plasma, a trait shared by Eritoran.

Blood-brain barrier (BBB) penetration is minimal for all compounds (BBB <0.3), which is advantageous for avoiding central nervous system (CNS)-related side effects, especially for non-CNS targets.

### Metabolism

Cytochrome P450 inhibition profiles suggest potential drug-drug interaction risks. All three candidates inhibit CYP1A2, with CID5403474 showing the highest score (1.0). CID74977829 notably inhibits CYP2C9 (0.893), which may necessitate caution in co-administration. CYP3A4 inhibition is moderate across all compounds (0.511–0.615), but Eritoran shows no inhibition, making it safer from a metabolic interaction perspective.

### Excretion

Plasma clearance (CLplasma) varies widely, with CID5898023 showing the fastest clearance (7.595 mL/min/kg), and Eritoran the slowest (2.254 mL/min/kg). Rapid clearance may necessitate higher or more frequent dosing.

Half-life (T_1_/_2_) values also diverge: CID74977829 has the longest predicted half-life (2.421 h), while CID5403474 exhibits the shortest (0.646 h). Eritoran, despite its large size, shows the longest half-life (4.596 h), probably due to extensive plasma binding and slow metabolism.

Toxicological predictions are favorable for all compounds. None are predicted to be hepatotoxic, carcinogenic, or mutagenic. However, only CID5403474 and Eritoran are predicted to exhibit cytotoxicity. Ecotoxicity is noted in the same two compounds.

### Toxicity profile

The LD_50_ values indicate low acute toxicity: CID5898023 is the most toxic (LD_50_ = 2000 mg/kg), followed by CID5403474 (4000 mg/kg), while both CID74977829 and Eritoran show higher safety margins (LD_50_ = 5000 mg/kg) (Table 9). Among the three candidates, CID5898023 stands out as the most promising oral drug-like compound due to its favorable absorption, high bioavailability, good QED score, and acceptable metabolic and toxicity profiles. CID5403474 also shows strong potential, albeit with slightly lower solubility and half-life. CID74977829, while relatively safe, suffers from poor permeability and higher polarity. Eritoran, the reference ligand, is clearly unsuitable for oral delivery and presents several pharmaceutical limitations, reinforcing the value of the proposed candidates as viable alternatives.

**TABLE 9:** In silico toxicological profile and LD_50_ values of selected TLR4-targeting compounds.

CID	Hepatotoxicity	Carcinogenicity	Mutagenicity	Cytotoxicity	Ecotoxicity	LD50
74977829	Inactive	Inactive	Inactive	Inactive	Inactive	5000
5403474	Inactive	Inactive	Inactive	Inactive	Active	4000
5898023	Inactive	Inactive	Inactive	Inactive	Inactive	2000
ERITORAN	Inactive	Inactive	Inactive	Inactive	Active	5000

Hepatotoxicity, mutagenicity, cytotoxicity, and ecotoxicity, where “inactive” indicates no detected toxicity in these categories. LD50 values (lethal dose for 50% of test subjects, measured in mg/kg) suggest acute toxicity levels, with higher values indicating lower toxicity.

### Molecular dynamics simulation (MD)

Molecular docking provides an initial approximation of the ligand–receptor interaction, but it represents a static snapshot that does not fully account for the dynamic nature of biomolecular systems. Therefore, molecular dynamics (MD) simulations were performed to evaluate the stability and conformational behavior of the TLR4-ligand complexes over time under near-physiological conditions. This step was used to complement the docking results by examining the temporal evolution of the complexes and identifying persistent binding modes.

To ensure the reliability of the findings, the MD analysis was first carried out for the reference compound, which serves as a benchmark to compare the dynamic performance of the newly selected TLR4-targeting candidates. Key parameters such as the root-mean-square deviation (RMSD), root-mean-square fluctuation (RMSF), protein–ligand contact maps, and terminal flexibility were analyzed to assess structural stability, residue-level mobility, and interaction persistence throughout the simulation trajectory.

### Comparative RMSD analysis

The structural dynamics of the TLR4/MD-2 complexes with Eritoran, CID5403474, CID5898023, and CID74977829 were comparatively evaluated over the 100-ns MD simulations using protein and ligand RMSD profiles (Figure 8). Distinct dynamic behaviors were observed among the four systems. The Eritoran complex showed noticeable fluctuations in both protein and ligand RMSD, with the protein RMSD reaching approximately 6.5 Å and the ligand RMSD varying within approximately 5–9 Å. Despite these fluctuations, the trajectories remained within a relatively consistent range during the later stages of the simulation.

**FIGURE 8:**
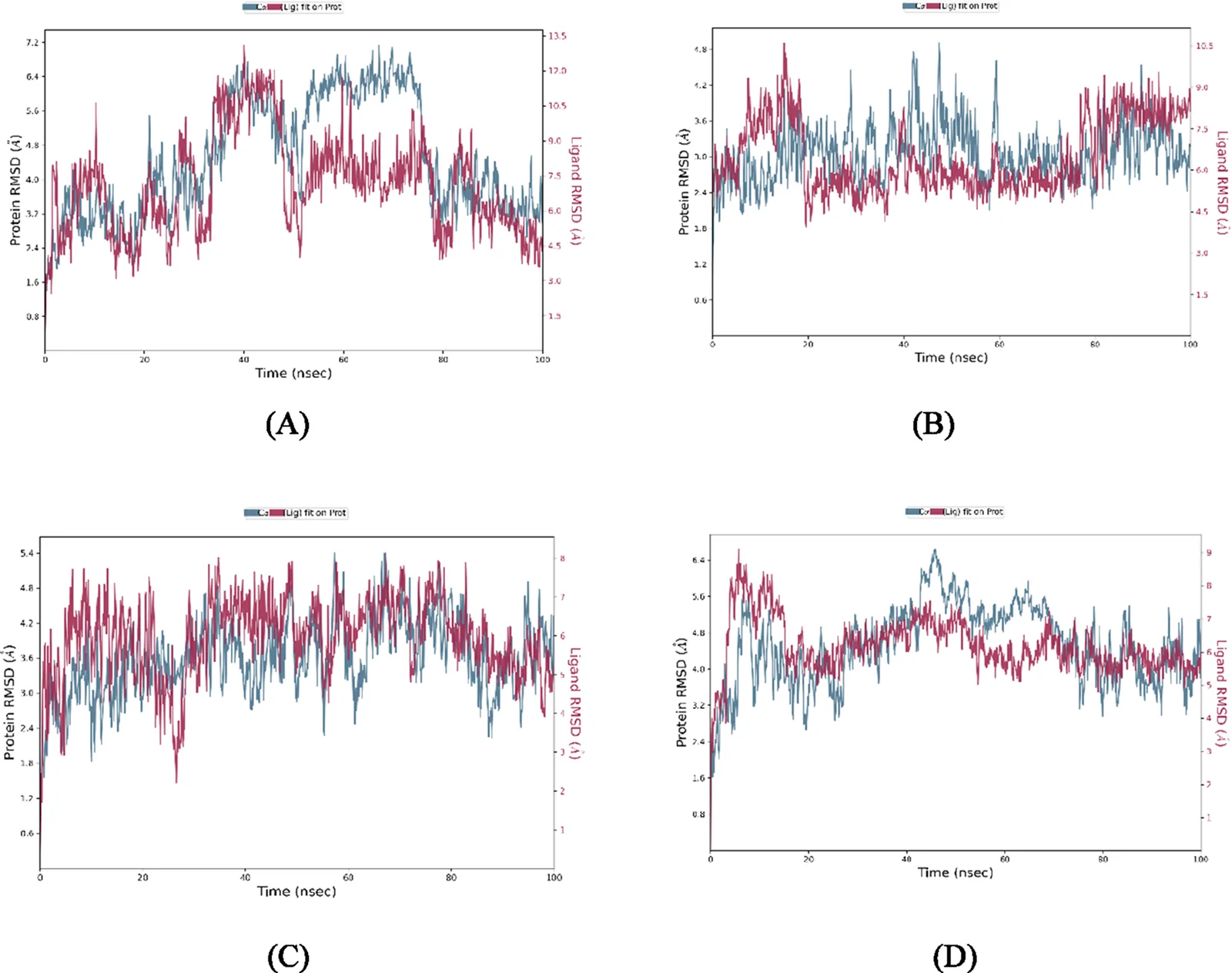
Comparative RMSD profiles of the TLR4/MD-2 complexes during 100-ns molecular dynamics simulations. Protein and ligand RMSD trajectories are shown for **(A)** Eritoran, **(B)** CID5403474, **(C)** CID5898023, and **(D)** CID74977829, illustrating differences in the conformational behavior of the four simulated systems.

CID5403474 exhibited the largest conformational variations among the tested compounds. Its ligand RMSD increased markedly during the trajectory, reaching approximately 13.5 Å, accompanied by substantial changes in the protein RMSD, which approached 7 Å. The pronounced transitions observed during the simulation suggest considerable ligand rearrangement relative to its initial docked configuration and indicate lower conformational stability of this complex compared with the other candidate systems.

In contrast, CID5898023 displayed a comparatively more restrained protein RMSD profile, generally fluctuating within approximately 2.4–4.8 Å. Although its ligand RMSD showed conformational adjustments within the binding environment, the magnitude of the protein backbone deviations was lower than that observed for CID5403474. CID74977829 similarly showed comparatively moderate protein RMSD fluctuations, approximately within the 2.5–4.8 Å range, while its ligand RMSD remained broadly within approximately 4–8 Å.

Overall, the comparative RMSD profiles indicate that CID5898023 and CID74977829 produced less pronounced protein backbone deviations than CID5403474 during the 100-ns simulations. CID5403474 showed the greatest conformational variability, particularly in the ligand trajectory, whereas Eritoran displayed an intermediate and fluctuating dynamic profile. These observations describe relative conformational behavior of the simulated complexes and should be interpreted together with RMSF and protein–ligand contact analyses rather than as standalone evidence of binding affinity.

### Comparative RMSF analysis

Residue-level flexibility of the TLR4/MD-2 complexes was comparatively assessed using the RMSF profiles of Eritoran, CID5403474, CID5898023, and CID74977829 (Figure 9). Overall, the four systems exhibited a broadly similar fluctuation pattern, characterized by relatively low RMSF values across most of the structured regions and increased mobility primarily in loop and terminal regions. For the majority of residues, fluctuations remained approximately below 2.5 Å, indicating that ligand-dependent differences were concentrated mainly in the more flexible regions of the protein rather than being distributed throughout the entire structure.

**FIGURE 9:**
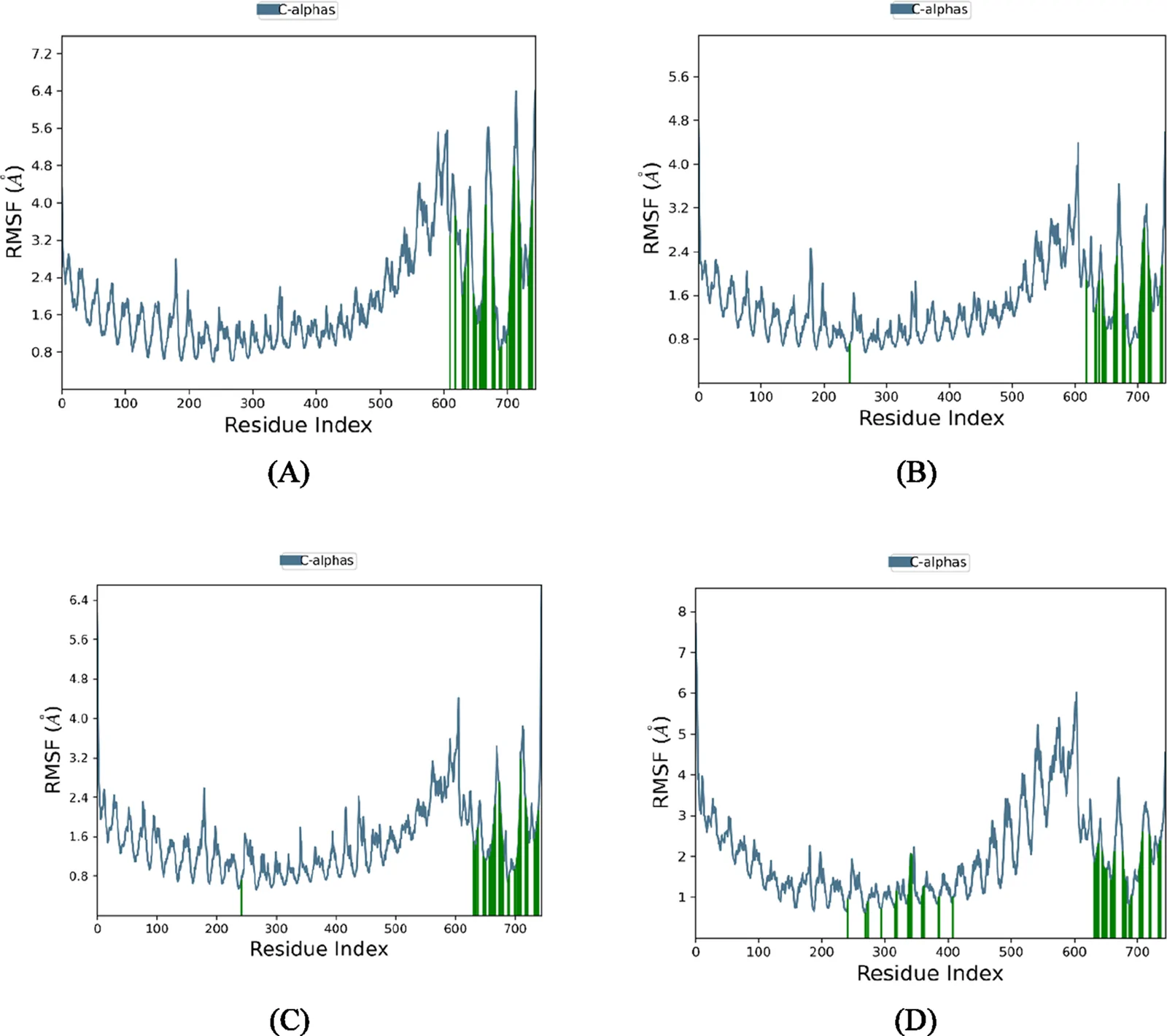
Comparative RMSF profiles of the TLR4/MD-2 complexes during the 100-ns molecular dynamics simulations. Residue-level fluctuations are shown for the complexes with **(A)** Eritoran, **(B)** CID5403474, **(C)** CID5898023, and **(D)** CID74977829. The profiles illustrate relatively limited fluctuations across most structured regions, with increased mobility predominantly localized to loop and terminal regions.

The Eritoran complex exhibited the largest localized fluctuations, with pronounced peaks in the later residue region reaching approximately 6–8 Å. CID5403474 showed a comparable increase in flexibility toward the C-terminal region, with several peaks reaching approximately 5–7 Å. These localized fluctuations were substantially higher than those observed throughout the central structured portion of the protein.

In comparison, the CID5898023 complex displayed a relatively restrained RMSF profile over most of the protein, with increased mobility occurring predominantly toward the terminal region, where the highest fluctuations approached approximately 4–5 Å. CID74977829 showed a similar pattern, with most residues maintaining relatively low fluctuations and localized increases toward the terminal region reaching approximately 4 Å.

Taken together, the RMSF profiles suggest that the overall residue-level flexibility pattern was conserved across the four simulated systems, while the magnitude of localized fluctuations differed among the complexes. Eritoran and CID5403474 displayed the largest local fluctuations, whereas CID5898023 and CID74977829 showed comparatively lower maximum deviations. Importantly, the major RMSF peaks were concentrated in flexible terminal and loop regions; therefore, these fluctuations should not by themselves be interpreted as evidence of destabilization of the ligand-binding region. The RMSF findings were consequently considered together with the RMSD trajectories and protein–ligand contact persistence analyses to characterize the relative dynamic behavior of the four complexes.

### Comparative protein–ligand interaction fraction analysis

The protein–ligand interaction histograms were comparatively analyzed to identify residues contributing most consistently to ligand recognition during the 100-ns simulations (Figure 10). Clear differences were observed among the four complexes in both the magnitude and distribution of interaction fractions. Eritoran displayed the most extensive interaction profile, with several residues contributing repeatedly throughout the trajectory. In particular, GLU92 and PHE104 showed prominent interaction fractions, with the cumulative contribution of GLU92 exceeding two in the interaction histogram, indicating that this residue participated in multiple interaction types during substantial portions of the simulation.

**FIGURE 10:**
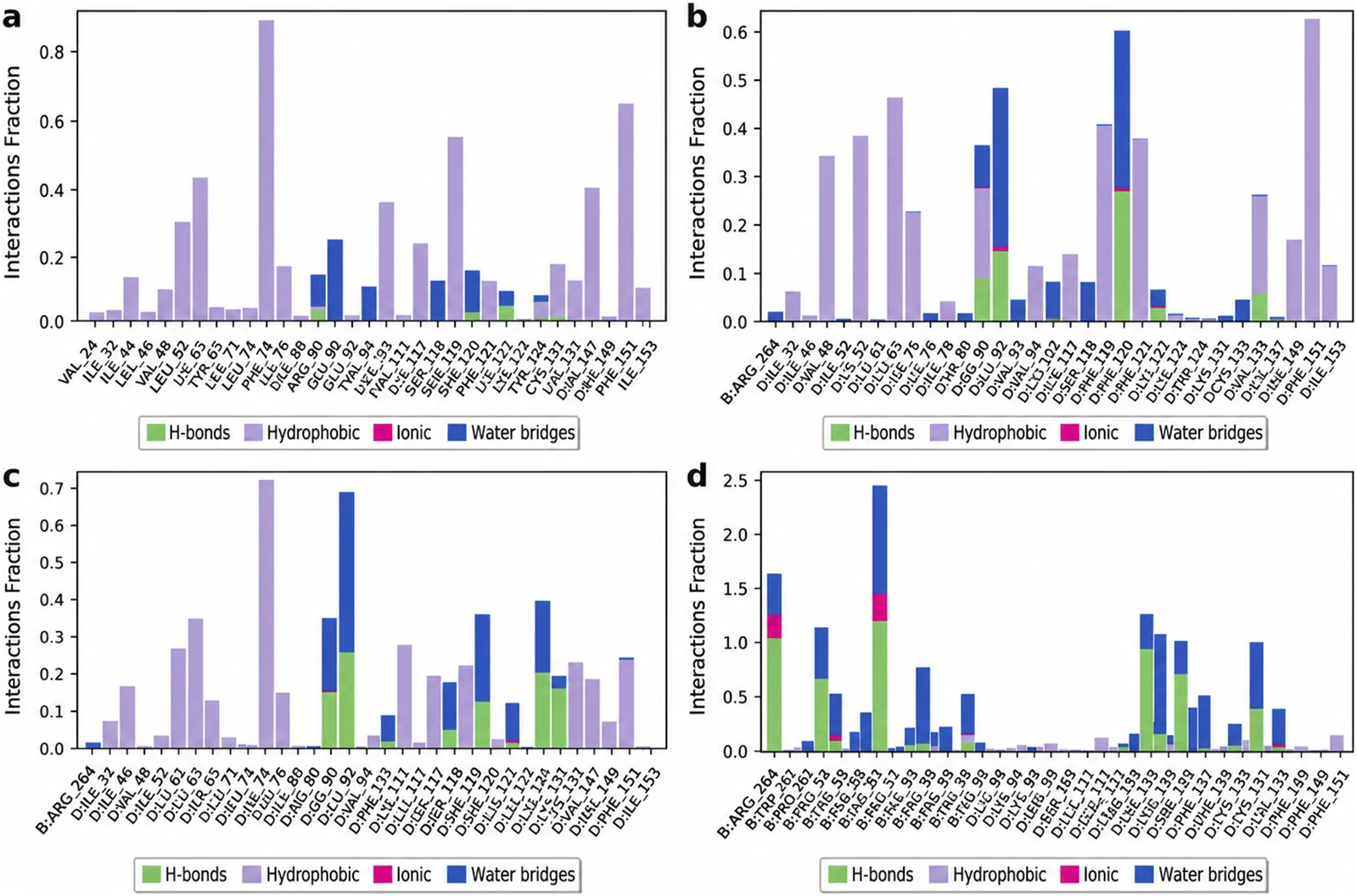
Comparative protein–ligand interaction fractions for the TLR4/MD-2 complexes during the 100-ns molecular dynamics simulations. Interaction fractions are shown for **(A)** Eritoran, **(B)** CID5403474, **(C)** CID5898023, and **(D)** CID74977829. The bars represent the fraction of simulation time during which individual residues participated in hydrogen-bond, hydrophobic, ionic, or water-mediated interactions with the ligand.

CID5403474 exhibited a more dispersed interaction pattern, with individual residue contributions generally lower than those observed for Eritoran. Although several residues participated in ligand contacts, no single residue showed comparably dominant interaction persistence, and the highest interaction fractions remained below approximately 0.9. This distribution is consistent with a more variable interaction network during the simulation.

CID5898023 showed a more focused set of interactions, with residues including GLU92, PHE121, PHE104, TYR114, SER119, and ILE153 contributing to ligand stabilization. Among these, GLU92 and PHE121 displayed some of the highest interaction fractions. Although their occupancies were lower than the most prominent contacts observed for Eritoran, the presence of recurrent contacts with several MD-2 pocket residues indicates persistent ligand–protein recognition during the trajectory.

CID74977829 also maintained interactions with several key residues, including GLU92, PHE121, PHE104, TYR114, VAL135, and ILE153. The interaction histogram revealed comparatively prominent contributions from PHE121 and GLU92, together with additional hydrophobic and polar contacts distributed across the binding pocket.

Overall, the interaction-fraction analysis showed that Eritoran maintained the densest and most persistent residue interaction network, whereas the three candidate compounds exhibited more selective interaction profiles. Among the candidates, CID5898023 and CID74977829 maintained recurrent contacts with several residues that also contributed to the reference complex, particularly GLU92 and PHE121. These results complement the RMSD and RMSF analyses by showing that differences among the complexes involve not only conformational fluctuations but also the persistence and distribution of protein–ligand contacts.

### Comparative protein–ligand contact timeline analysis

The protein–ligand contact timelines were comparatively examined to assess the temporal persistence and distribution of interactions throughout the 100-ns MD simulations (Figure 11). The four complexes exhibited distinct contact patterns, indicating differences in how individual ligands maintained interactions with the TLR4/MD-2 binding environment over time.

**FIGURE 11:**
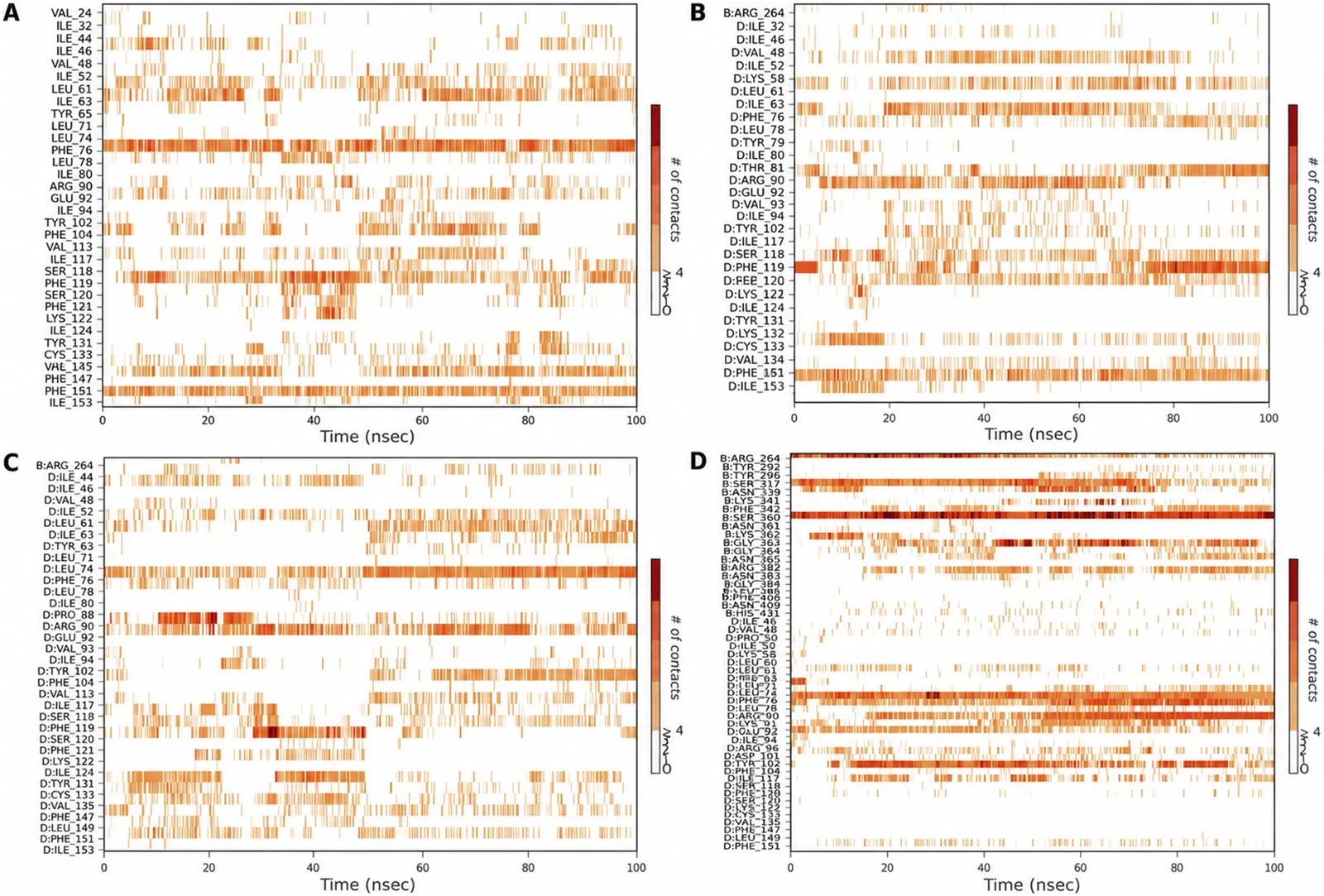
Comparative protein–ligand contact timelines for the TLR4/MD-2 complexes during the 100-ns molecular dynamics simulations. Contact timelines are shown for **(A)** Eritoran, **(B)** CID5403474, **(C)** CID5898023, and **(D)** CID74977829. Each row represents the temporal occurrence of contacts between the ligand and individual protein residues throughout the simulation trajectory, allowing comparison of interaction persistence and recurrence among the four complexes.

Eritoran displayed the most extensive contact pattern, with multiple residue interactions distributed across a substantial portion of the simulation trajectory. Several contacts persisted or repeatedly re-formed throughout the 100-ns simulation, producing relatively continuous interaction bands in the timeline. This pattern indicates that the reference ligand maintained a broad interaction network despite the conformational fluctuations observed in its RMSD profile.

In comparison, CID5403474 exhibited a more intermittent contact pattern. Several interactions appeared only during restricted portions of the trajectory, while others were repeatedly formed and disrupted. The lower temporal continuity of these contacts is consistent with the pronounced ligand RMSD variations observed for this complex and suggests substantial rearrangement of the ligand–protein interaction network during the simulation.

CID5898023 showed recurrent contacts distributed across the trajectory, although the interaction pattern was less dense than that observed for Eritoran. Several residues repeatedly interacted with the ligand at different stages of the simulation, indicating that protein–ligand contacts were re-established despite conformational adjustments. This behavior is consistent with its comparatively restrained protein RMSD and RMSF profiles.

CID74977829 likewise exhibited recurrent protein–ligand contacts over the 100-ns trajectory. Multiple interaction bands were observed across different simulation intervals, indicating repeated engagement of the ligand with residues within the binding environment. Although the contact network was less extensive than that of Eritoran, its temporal distribution was more sustained than the highly intermittent pattern observed for CID5403474.

Overall, the contact timeline analysis complements the interaction-fraction results by demonstrating that Eritoran maintained the most extensive temporal interaction network, whereas the three candidate compounds displayed more selective and dynamic contact patterns. Among the candidates, CID5898023 and CID74977829 showed recurrent interactions distributed across the simulation, while CID5403474 exhibited greater temporal variability. When considered together with the RMSD and RMSF profiles, these findings indicate distinct dynamic behaviors among the four complexes without, by themselves, establishing differences in binding affinity or pharmacological activity.

### Quantitative analysis of persistent protein–ligand interactions during MD simulations

Quantitative occupancy analysis further revealed distinct interaction-persistence patterns among the four complexes (Supplementary Table S3). CID5403474 showed minimal hydrogen-bond persistence and was predominantly characterized by hydrophobic contacts, particularly PHE76 (47.45%), ILE63 (38.76%), and PHE147 (33.67%). CID5898023 displayed a mixed interaction profile, with moderate hydrogen-bond occupancy involving SER120 (18.78%) and GLU92 (10.59%), together with persistent hydrophobic contacts involving PHE151 (44.66%), PHE76 (36.46%), LEU61 (32.27%), and ILE52 (32.07%). CID74977829 similarly exhibited contributions from both interaction types, with GLU92 showing the highest hydrogen-bond occupancy (23.98%) and PHE76 the highest hydrophobic occupancy (32.17%). In contrast, Eritoran exhibited a pronounced persistent hydrogen-bond network, dominated by GLU92 (93.81%), LYS362 (83.42%), and ASN339 (70.43%), whereas its individual hydrophobic contacts showed substantially lower occupancies. Overall, these results indicate distinct interaction-persistence profiles, with the candidate compounds relying predominantly on recurrent hydrophobic contacts to varying degrees, while Eritoran was characterized by extensive persistent hydrogen bonding. Detailed residue-level occupancies are provided in Supplementary Table S3.

### Proposed experimental validation of the prioritized compounds

To translate the computational predictions into experimentally testable hypotheses, a stepwise *in vitro* validation strategy is proposed for the three prioritized compounds. First, direct binding to recombinant MD-2 should be evaluated using surface plasmon resonance (SPR), allowing determination of binding affinity and kinetic parameters and comparison with the reference TLR4/MD-2 antagonist Eritoran. Compounds showing measurable MD-2 binding would subsequently be evaluated in a TLR4 reporter cell system. Cells would be stimulated with lipopolysaccharide (LPS) to activate TLR4/MD-2 signaling and treated with the selected compounds at a range of non-cytotoxic concentrations. Eritoran would serve as a reference antagonist, while LPS-stimulated and unstimulated cells would provide positive and basal controls, respectively. Reduction of the TLR4-dependent reporter signal relative to the LPS-treated control would provide functional evidence of pathway modulation.

Compounds demonstrating activity in the reporter assay would then be evaluated in an appropriate LPS-responsive immune cell model to determine their effects on inflammatory mediator production. IL-6 and TNF-α levels in the culture supernatant would be quantified following LPS stimulation in the presence or absence of the selected compounds. Cell viability should be assessed in parallel to distinguish genuine anti-inflammatory effects from reductions in cytokine production caused by cytotoxicity. Together, direct MD-2 binding by SPR, modulation of TLR4-dependent signaling, and reduction of LPS-induced IL-6 and TNF-α production would provide complementary biophysical and functional evidence for experimental validation of the computationally prioritized compounds. Subsequent studies could extend this validation to infection-relevant cellular models and, if supported by the *in vitro* findings, appropriate *in vivo* models.

## Discussion

### Study novelty

The principal novelty of this study lies in the integration of a tripartite host–virus–bacterium network with structure-based computational screening to investigate TLR4/MD-2 in the context of SARS-CoV-2–*Acinetobacter baumannii* co-infection. Rather than selecting TLR4/MD-2 *a priori* as an isolated inflammatory target, the workflow integrated SARS-CoV-2, *A. baumannii*, and human immune-associated proteins within a unified protein–protein interaction network, followed by network topology and functional enrichment analyses to identify biologically relevant host-associated targets. TLR4 was subsequently prioritized for structure-based investigation based on its network relevance, mechanistic association with LPS-mediated inflammatory signaling, availability of a structurally characterized TLR4/MD-2 ligand-binding cavity, and availability of Eritoran as a reference antagonist.

A further distinguishing feature of the study is the sequential integration of network analysis with molecular docking, ADMET and toxicity prediction, 100-ns molecular dynamics simulations, and quantitative evaluation of protein–ligand interaction persistence. This multilevel computational framework enables candidate prioritization based on complementary structural, dynamic, and predicted pharmacokinetic criteria rather than docking scores alone. The novelty therefore resides in linking inter-species network analysis of a viral–bacterial co-infection model with downstream structural prioritization of TLR4/MD-2 and comparative dynamic assessment of candidate ligands, rather than in identifying TLR4/MD-2 as a previously unknown pharmacological target.

### Scope, translational interpretation, and experimental validation

The findings of this study should be interpreted within the scope of computational lead screening. Although the integrated analyses identified compounds displaying favorable predicted binding, ADMET profiles, and dynamic behavior within the TLR4/MD-2 binding site, these observations represent computational predictions rather than experimentally demonstrated pharmacological activity. Accordingly, the identified compounds should be regarded as prioritized candidates for further investigation rather than validated TLR4/MD-2 inhibitors. Confirmation of their biological relevance will require experimental assessment of direct MD-2 binding and TLR4-mediated inflammatory signaling, followed by appropriate cellular and, ultimately, *in vivo* validation.

### Network-based identification and biological relevance of TLR4

Topological analysis of the integrated SARS-CoV-2–*A. baumannii*–human interaction network identified a set of prioritized proteins across the three biological components. Among the host proteins emerging from this analysis, TLR4 was subsequently prioritized for structure-based investigation by considering its network position together with its biological relevance to co-infection-associated inflammatory signaling. In particular, its established role in LPS-mediated innate immune responses provided a strong biological rationale for further investigation (Feng et al., 2024).

The hub analysis identified inter-species proteins comprising human host, SARS-CoV-2, and *A. baumannii* components, reflecting the integrated nature of the tripartite network. These proteins therefore represent highly connected nodes across the host–virus–bacterium interaction landscape rather than exclusively human immune hubs. The occurrence of pathogen-derived and host-associated proteins among the highly ranked nodes indicates that network centrality in the modeled co-infection system is distributed across multiple biological components. Within this inter-species context, human proteins such as TLR4 and IL6 are particularly relevant because of their association with host inflammatory and immune-response pathways.

The integrated network represents the molecular landscape of co-infection without explicitly modeling its temporal progression. In clinical settings, secondary bacterial infection may develop after the initial viral infection. Consequently, the interactions represented by the network should be interpreted as an integrated representation of potentially relevant molecular relationships rather than as a time-resolved sequence of infection events.

Functional enrichment analyses further supported the biological relevance of TLR4-associated inflammatory signaling, highlighting pathways including Toll-like receptor signaling and NF-κB activation, both of which have been implicated in dysregulated inflammatory responses during severe infections (Aikawa et al., 2012). Although additional proteins, including IL6, HSP90AB1, and HSP90B1, were identified as relevant through the network and enrichment analyses, TLR4 was prioritized for the structure-based component using combined biological and structural criteria. Its involvement in LPS-mediated inflammatory signaling during bacterial co-infection, the presence of a structurally characterized ligand-binding cavity within the TLR4/MD-2 complex, and the availability of Eritoran as a reference antagonist provided a consistent basis for molecular docking and subsequent molecular dynamics analysis.

Although the integrated network also contained viral and bacterial proteins, the structure-based component was intentionally restricted to a human host target because the objective of this stage was to explore host-directed modulation of co-infection-associated inflammatory signaling rather than direct antiviral or antibacterial inhibition. Viral and bacterial proteins were therefore retained as essential components of the tripartite network for characterizing the cross-species molecular context but were not subjected to docking or MD simulations in the present study. Systematic evaluation of pathogen-derived targets would represent a complementary direct antiviral or antibacterial strategy and was beyond the scope of the current structure-based screening. Future studies could extend this framework to selected viral and bacterial targets to evaluate complementary multi-target intervention strategies. Thus, network centrality alone was not used as the criterion for structure-based target selection.

### Structural interpretation of the predicted binding modes

Following network-based target prioritization, molecular docking was used to examine the predicted accommodation of the selected compounds within the TLR4/MD-2 binding cavity. The three candidate compounds produced more favorable docking scores than the reference antagonist Eritoran, with CID5898023 displaying the most favorable predicted docking score. Residue-level analysis indicated that the compounds were accommodated within the predominantly hydrophobic MD-2 pocket through interactions involving PHE76, PHE104, ILE46, ILE61, ILE63, ILE94, ILE117, and VAL135. PHE76 was recurrently involved across the selected candidates, while ligand-specific aromatic contacts involving PHE147 and PHE151 were also observed. These interactions provide a structural rationale for the predicted docking poses and indicate accommodation of the selected compounds within the MD-2 binding cavity. Nevertheless, molecular docking represents a static computational approximation and does not establish experimental binding affinity or TLR4/MD-2 antagonistic activity.

### Predicted ADMET and toxicity profiles

ADMET and toxicity predictions provided an additional computational layer for comparing the prioritized candidates. CID5898023 showed favorable predicted absorption, bioavailability, and drug-likeness characteristics; however, its comparatively lower predicted LD_50_ indicates a potential toxicity concern requiring experimental assessment rather than evidence of an acceptable clinical safety margin. CID74977829 also showed a generally favorable predicted profile, although its predicted absorption was lower.

Eritoran presented a markedly different physicochemical and pharmacokinetic profile. Its high molecular weight and established intravenous administration profile represent important considerations for its pharmaceutical development (Aikawa et al., 2012). In comparison, the smaller natural-product-derived scaffolds prioritized in the present screening may provide physicochemical features suitable for subsequent optimization. Nevertheless, these predicted characteristics constitute computational prioritization criteria and should not be interpreted as evidence of improved clinical developability.

### Molecular dynamics and interaction persistence

Molecular dynamics simulations provided further insight into the conformational behavior of the four TLR4/MD-2 complexes over the 100-ns trajectories. The systems exhibited distinct dynamic profiles. CID5403474 showed the largest ligand conformational variations, whereas CID5898023 and CID74977829 displayed comparatively more restrained protein-level fluctuations. The RMSD and RMSF profiles were interpreted together with the interaction-fraction and contact-timeline analyses to characterize relative dynamic behavior rather than as independent measures of binding affinity.

Quantitative analysis of protein–ligand contact occupancy further revealed substantial differences in interaction persistence among the simulated complexes. CID5403474 exhibited minimal hydrogen-bond persistence and was characterized predominantly by recurrent hydrophobic interactions, particularly involving PHE76, ILE63, and PHE147. CID5898023 displayed a mixed interaction profile, combining moderate hydrogen-bond persistence with several recurrent hydrophobic contacts, including PHE151 and PHE76. CID74977829 similarly exhibited contributions from both hydrogen-bond and hydrophobic interactions. In contrast, Eritoran was characterized by a pronounced persistent hydrogen-bond network, particularly involving GLU92, whereas its individual hydrophobic contacts showed lower occupancies. These quantitative findings complement the RMSD, RMSF, interaction-fraction, and contact-timeline analyses and demonstrate distinct modes of interaction persistence among the four simulated systems.

### Integrated interpretation and future experimental validation

Considering the computational evidence collectively, CID5898023 exhibited a comparatively balanced predicted profile across docking, ADMET, toxicity, and MD-derived interaction analyses, while CID74977829 also retained characteristics supporting its prioritization for further investigation. CID5403474 produced a favorable docking score but displayed greater conformational variability during the MD simulation, illustrating that the initial docking score alone did not fully reflect subsequent dynamic behavior. These observations emphasize the value of integrating multiple computational criteria rather than prioritizing candidate compounds solely according to docking scores.

Importantly, none of these computational findings demonstrates TLR4/MD-2 antagonism or therapeutic efficacy. Experimental validation should therefore include direct assessment of ligand–MD-2 binding, such as by surface plasmon resonance, together with TLR4 reporter assays and quantification of downstream pro-inflammatory mediators such as IL-6 and TNF-α under appropriate stimulation conditions. Subsequent cellular and *in vivo* investigations would be required before pharmacological or therapeutic conclusions could be established. Within these limitations, the integrated network-informed and structure-based workflow provides a computational framework for prioritizing candidate compounds for subsequent experimental evaluation in host-directed inflammatory research.

## Conclusion

This study investigated the molecular interplay underlying *SARS-CoV-2/A. baumannii* co-infection through an integrated host–virus–bacterium interactome, identifying TLR4 as a highly ranked host immune-associated node. In silico screening against the TLR4/MD-2 complex prioritized three candidates: CID5898023 (curcumin-derived), CID74977829 (baicalin-derived), and CID5403474 (chrysin-derived). Among them, CID5898023 showed the most favorable overall computational profile, combining the strongest docking score with favorable predicted ADMET characteristics and comparatively restrained dynamic behavior during MD simulations. CID74977829 also showed characteristics supporting its prioritization, whereas CID5403474 exhibited greater conformational variability despite its favorable docking score. Collectively, these computational findings prioritize CID5898023 and CID74977829 for subsequent experimental evaluation of their interaction with TLR4/MD-2 and potential modulation of TLR4-mediated inflammatory signaling. Experimental validation is required before any pharmacological or therapeutic conclusions can be established.

## Data Availability

The network datasets and centrality-analysis outputs generated in this study, including the integrated protein–protein interaction network and the Top-30 917 node rankings obtained using six cytoHubba algorithms (MCC, Degree, MNC, DMNC, Bottleneck, and Closeness), are publicly available in the Zenodo repository at DOI: 10.5281/zenodo.21891360. Additional data supporting the findings of this study are available from the corresponding author upon reasonable request.
