# Supplementary material for: Identification of natural TLR4 modulators through network pharmacology and molecular modeling in SARS-CoV-2 Acinetobacter baumannii co-infection
Source: Front Chem. 2026 Sep 16;14:1916568. doi: 10.3389/fchem.2026.1916568 (PMC13625435; doi:10.3389/fchem.2026.1916568)
Supplement: Supplementary file 1 [file DataSheet1.docx]

**SUPPLEMENTARY FILE**

**Identification of Natural TLR4 Modulators Through Network Pharmacology and Molecular Modeling in SARS-CoV-2/A. baumannii Co-Infection**

Mebarka OUASSAF^1^*, Shafi Ullah KHAN^2,3^, Kannan R R RENGASAMY^4,5^, Nada S. ALHAGGASS^6^, and Bader Y. ALHATLANI^7^*

^1^Group of Computational and Medicinal Chemistry, LMCE Laboratory, University of Biskra, Biskra (07000), Algeria; [nouassaf@univ-biskra.dz](mailto:nouassaf@univ-biskra.dz)

^2^ Inserm U1086 ANTICIPE (Interdisciplinary Research Unit for Cancer Prevention and Treatment), Normandie Université, Universite de Caen Normandie, 14076 Caen, France; [shafiullahpharmd@gmail.com](mailto:shafiullahpharmd@gmail.com)

^3^	Comprehensive Cancer Center Francois Baclesse, UNICANCER, 14076 Caen, France

^4^	Laboratory of Natural Products and Medicinal Chemistry (LNPMC), Saveetha Medical College and Hospital, Saveetha Institute of Medical and Technical Sciences (SIMATS), Thandalam, Chennai 602105, India; [kannan@LNPMC.in](mailto:kannan@LNPMC.in)

^5^	Centre of Excellence for Pharmaceutical Sciences, North-West University, Potchefstroom 2520, South Africa

^6^ Department of Biology, College of Science, Qassim University, Saudi Arabia; [ndsahs@qu.edu.sa](mailto:ndsahs@qu.edu.sa)

^7^ Unit of Scientific Research, Applied College, Qassim University, Buraydah 52571, Saudi Arabia; [balhatlani@qu.edu.sa](mailto:balhatlani@qu.edu.sa)

***** Correspondence: [balhatlani@qu.edu.sa](mailto:balhatlani@qu.edu.sa) (B.Y.A.); [nouassaf@univ-biskra.dz](mailto:nouassaf@univ-biskra.dz) (M.O.)

**Table S1. Multi-metric comparison of hub proteins identified by six cytoHubba centrality algorithms.**

| **Protein** | **MCC** | **Degree** | **MNC** | **Bottleneck** | **DMNC** | **Closeness** | **Consensus** |
| --- | --- | --- | --- | --- | --- | --- | --- |
| ORF10 | ✓ | ✓ | ✓ | ✓ | — | ✓ | **5/6** |
| ORF7a | ✓ | ✓ | ✓ | ✓ | — | ✓ | **5/6** |
| ORF8 | ✓ | ✓ | ✓ | ✓ | — | ✓ | **5/6** |
| nsp13 | ✓ | ✓ | ✓ | ✓ | — | ✓ | **5/6** |
| nsp3 | ✓ | ✓ | ✓ | ✓ | — | ✓ | **5/6** |
| nsp8 | ✓ | ✓ | ✓ | ✓ | — | ✓ | **5/6** |
| nsp9 | ✓ | ✓ | ✓ | ✓ | — | ✓ | **5/6** |
| E | — | ✓ | ✓ | ✓ | — | ✓ | **4/6** |
| HSP90B1 | ✓ | — | ✓ | — | ✓ | ✓ | **4/6** |
| M | — | ✓ | ✓ | ✓ | — | ✓ | **4/6** |
| N | — | ✓ | ✓ | ✓ | — | ✓ | **4/6** |
| ORF3b | ✓ | ✓ | ✓ | — | — | ✓ | **4/6** |
| ORF6 | — | ✓ | ✓ | ✓ | — | ✓ | **4/6** |
| ORF7b | — | ✓ | ✓ | ✓ | — | ✓ | **4/6** |
| ORF9b | — | ✓ | ✓ | ✓ | — | ✓ | **4/6** |
| S | — | ✓ | ✓ | ✓ | — | ✓ | **4/6** |
| nsp1 | — | ✓ | ✓ | ✓ | — | ✓ | **4/6** |
| nsp10 | ✓ | ✓ | ✓ | — | — | ✓ | **4/6** |
| nsp12 | ✓ | ✓ | ✓ | — | — | ✓ | **4/6** |
| nsp15 | ✓ | ✓ | ✓ | ✓ | — | — | **4/6** |
| nsp16 | — | ✓ | ✓ | ✓ | — | ✓ | **4/6** |
| nsp3ab | — | ✓ | ✓ | ✓ | — | ✓ | **4/6** |
| nsp6 | ✓ | ✓ | ✓ | — | — | ✓ | **4/6** |
| nsp7 | ✓ | ✓ | ✓ | — | — | ✓ | **4/6** |
| AP2M1 | — | — | ✓ | — | ✓ | ✓ | **3/6** |
| CANX | — | ✓ | ✓ | — | — | ✓ | **3/6** |
| IL6 | ✓ | — | — | ✓ | ✓ | — | **3/6** |
| ORF1ab | — | ✓ | ✓ | — | — | ✓ | **3/6** |
| PPIA | — | ✓ | ✓ | — | — | ✓ | **3/6** |
| nsp4 | — | ✓ | ✓ | — | — | ✓ | **3/6** |
| 1096995.BJAB07104_02054 | ✓ | — | — | ✓ | — | — | **2/6** |
| DDX21 | — | — | — | — | ✓ | ✓ | **2/6** |
| HSP90AA1 | — | ✓ | — | — | ✓ | — | **2/6** |
| HSP90AB1 | ✓ | — | — | — | ✓ | — | **2/6** |
| ORF14 | — | ✓ | ✓ | — | — | — | **2/6** |
| SND1 | — | ✓ | — | — | ✓ | — | **2/6** |
| **TLR4** | ✓ | — | — | ✓ | — | — | **2/6** |

Table S2. Significantly enriched KEGG pathways identified by DAVID 2026 among the selected human hub proteins.

| **KEGG pathway** | **Count** | **Fold enrichment** | **P-value** | **FDR** | **Associated genes** |
| --- | --- | --- | --- | --- | --- |
| Salmonella infection | 5 | 23.63 | 1.57×10⁻⁵ | 0.00116 | IL6, HSP90AB1, ARPC3, TLR4, HSP90B1 |
| NOD-like receptor signaling pathway | 4 | 25.07 | 2.58×10⁻⁴ | 0.00953 | IL6, HSP90AB1, STING1, TLR4 |
| Lipid and atherosclerosis | 4 | 21.70 | 3.94×10⁻⁴ | 0.00973 | IL6, HSP90AB1, TLR4, HSP90B1 |
| Shigellosis | 4 | 18.53 | 6.27×10⁻⁴ | 0.01160 | STING1, ARPC3, TLR4, SQSTM1 |
| PI3K-Akt signaling pathway | 4 | 12.95 | 1.78×10⁻³ | 0.02490 | IL6, HSP90AB1, TLR4, HSP90B1 |
| IL-17 signaling pathway | 3 | 37.40 | 2.02×10⁻³ | 0.02490 | IL6, HSP90AB1, HSP90B1 |
| Yersinia infection | 3 | 25.48 | 4.30×10⁻³ | 0.04210 | IL6, ARPC3, TLR4 |
| Fluid shear stress and atherosclerosis | 3 | 24.76 | 4.55×10⁻³ | 0.04210 | HSP90AB1, SQSTM1, HSP90B1 |

Table S3. Quantitative occupancy of major protein–ligand interactions during the 100-ns MD simulations

| **Complex** | **Hydrogen-bond contacts** | **Occupancy (%)** | **Hydrophobic contacts** | **Occupancy (%)** |
| --- | --- | --- | --- | --- |
| **CID5403474** | LYS122 | 3.20 | **PHE76** | **47.45** |
|  | ARG90 | 2.50 | **ILE63** | **38.76** |
|  | SER120 | 1.80 | **PHE147** | **33.67** |
|  | — | — | PHE151 | 28.57 |
|  | — | — | LEU61 | 27.27 |
| **CID5898023** | SER120 | 18.78 | **PHE151** | **44.66** |
|  | GLU92 | 10.59 | **PHE76** | **36.46** |
|  | CYS133 | 3.20 | **LEU61** | **32.27** |
|  | ARG90 | 3.10 | **ILE52** | **32.07** |
|  | — | — | PHE121 | 22.28 |
| **CID74977829** | GLU92 | 23.98 | **PHE76** | **32.17** |
|  | TYR131 | 19.18 | ILE63 | 28.17 |
|  | CYS133 | 14.39 | LEU61 | 23.98 |
|  | SER120 | 11.99 | VAL135 | 20.78 |
|  | ARG90 | 11.79 | ILE117 | 18.48 |
| **Eritoran** | **GLU92** | **93.81** | PHE76 | 12.39 |
|  | **LYS362** | **83.42** | PHE151 | 10.89 |
|  | **ASN339** | **70.43** | PHE119 | 9.79 |
|  | **ARG96** | **46.35** | PHE408 | 7.49 |
|  | **ARG264** | **43.96** | ILE94 | 6.19 |
|  | **SER120** | **40.96** | — | — |

Figure S1. Overview of the full integrated protein–protein interaction network prior to hub prioritization. The network comprises 218 nodes and 4,713 edges representing the integrated SARS-CoV-2, A. baumannii, and human host interaction network. Owing to the high network density, the figure is provided as a global representation of network connectivity; the complete network files are available in the associated Zenodo repository for interactive exploration and reproducibility.
